# Contrasting drivers of South Asian summer and winter monsoon evolution during the last deglaciation

**DOI:** 10.1038/s41561-026-02023-z

**Published:** 2026-06-22

**Authors:** Igor Obreht, Andreas Lückge, Mahyar Mohtadi, Petra Zahajská, Enno Schefuß, Denis Scholz, Lars Wörmer, Petter Hällberg, Alexander Budsky, Florian Adolphi, Gerald H. Haug, Martin Grosjean, Kai-Uwe Hinrichs

**Affiliations:** 1 MARUM – Center for Marine Environmental Sciences, University of Bremen, Bremen, Germany; 2 Institute of Geosciences, Johannes Gutenberg University Mainz, Mainz, Germany; 3 BGR– Federal Institute for Geosciences and Natural Resources, Hannover, Germany; 4 Faculty of Geosciences, University of Bremen, Bremen, Germany; 5 Institute of Geography and Oeschger Center for Climate Change Research, University of Bern, Bern, Switzerland; 6 Department of Earth Sciences, Uppsala University, Uppsala, Sweden; 7 Department of Geosciences, Landesmuseum für Kärnten, Klagenfurt, Austria; 8 Alfred Wegener Institute, Helmholtz Centre for Polar and Marine Research, Bremerhaven, Germany; 9 Department of Climate Geochemistry, Max Planck Institute for Chemistry, Mainz, Germany; 10 Department of Earth and Planetary Sciences, ETH Zürich, Zurich, Switzerland

**Keywords:** Palaeoclimate, Atmospheric dynamics

## Abstract

Reconstructing past South Asian monsoon dynamics, which governs wind and rainfall patterns across the Indian subcontinent, is crucial for understanding low-latitude climate. Constraining monsoon drivers requires separating summer and winter components in palaeoclimate archives, but this remains challenging because proxy signals usually integrate seasonal signals. The northeastern Arabian Sea provides a unique setting in which sedimentary and geochemical proxies are driven by the summer monsoon, while sea surface temperature is primarily controlled by winter monsoon winds. Here we employed mass spectrometry and hyperspectral imaging on a sediment core off Pakistan to reconstruct subdecadal changes in sea surface temperature and marine primary production during the last deglaciation (~16,000–12,000 years ago). Atmospheric humidity and vegetation changes were additionally assessed by plant-wax isotope analyses on a subset of samples. We show that summer monsoon winds were driven by the Northern Hemisphere high-latitude climate on centennial-to-millennial timescales. Winter monsoon was primarily characterized by a millennial-scale decline in wind strength driven by increasing global temperatures, with superimposed centennial-scale variability. We identified an inverse relationship between winter monsoon wind strength and the amount of winter non-monsoonal precipitation. Mechanistic insights of seasonal monsoon dynamics improved interpretation of regional palaeoprecipitation records and may enhance climate model performances in low latitudes.

## Abstract (web summary)

The intensity and centennial-scale variability of the winter and summer South Asian monsoons were controlled by different drivers during the last deglaciation, according to mass spectrometry and hyperspectral imaging, supported by geochemical analyses of a sediment core from the Arabian Sea.

## Main

Coupled land–ocean–atmosphere interactions play a key role in climate variability across various temporal scales. Among those interactions, monsoons stand out as crucial phenomena in low latitudes. The South Asian monsoon (SAM) profoundly affects the livelihoods of almost two billion people on the Indian subcontinent^1^. The South Asian summer monsoon (SASM) is thermally forced by the sensible heating of northern India that favours the occurrence of convection north of the Equator, and the seasonal migration of the Intertropical Convergence Zone (ITCZ)^2^. The strong southwesterly winds and heavy rainfall over South Asia during summer fuel marine primary production in the Arabian Sea. By contrast, the South Asian winter monsoon (SAWM) features weaker, drier northeasterly winds, resulting in reduced rainfall. An important limitation hampering our comprehension of the SAM system under a changing climate is that palaeoclimate reconstruction predominantly revolves around SASM, characterized by intense rainfall and upwelling-fuelled increased primary production that typically dominate proxy records^3–5^. Consequently, our understanding of SAWM dynamics under different boundary conditions remains limited, impeding a comprehensive grasp of the SAM system.

Examining abrupt climate transitions in the past provides valuable insights into the response of the SAM to rapid climate change. The last deglaciation occurred within a distinct climatic background characterized by increasing Northern Hemisphere summer insolation, the progressive retreat of continental ice sheets, and high-amplitude millennial-scale climate variability. The rapid warming at the onset of the Greenland Interstadial (GI) 1 at ~14,690 years before 2000 (b2k), and pronounced cooling associated with the Greenland Stadial (GS) 1 in the Northern Hemisphere at ~12,890 years b2k are two of the most recent abrupt climate transitions^6^. Consequently, the last deglaciation represents an ideal natural experiment for elucidating the evolution of the SAM system amidst major climate changes.

**Fig. 1:**
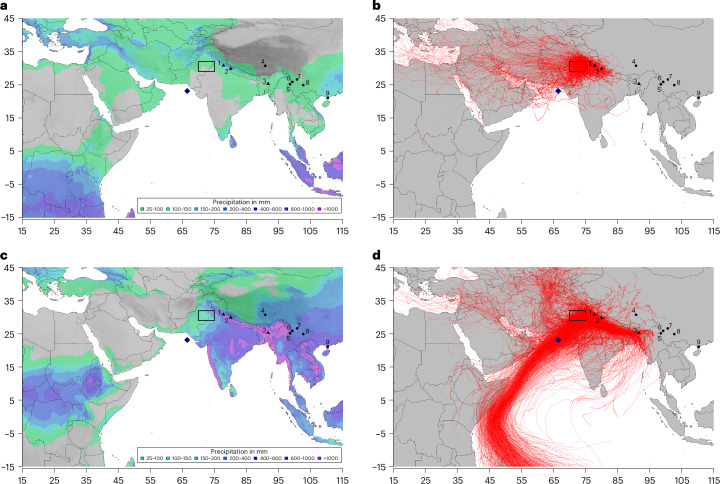
Core location and precipitation regimes during the summer and winter monsoon seasons. **a**, Plot of the winter (December, January, February) precipitation by WorldClim 2 (ref. ^53^). **b**, Back trajectories for 240 h during winter. **c**, Plot of the summer (June, July, August) precipitation by WorldClim 2 (ref. ^53^). **d**, Back trajectories for 240 h during summer. In all panels, longitude and latitude are shown in degrees (°) and the core location is indicated by a dark-blue diamond, where the rectangle displays the region for the calculation of the trajectories in **b** and **d**. This site was selected as representative of the middle Indus catchment and because of its proximity to the Timata and Bitto caves. Other sites discussed in this study are indicated by numbers (caves: black triangles; lakes: black circles): 1, Bittoo Cave; 2, Timta Cave; 3, Mawmluh Cave; 4, Lake Nam Co; 5, Lake Tengchongqingha; 6, Lake Tianchi; 7, Lake Chenghai; 8, Lake Dian; 9, Huguang Maar.

Here we used sediment core SO130-289KL (23°07.34′ N, 66°29.84′ E; 571 m water depth) off the coast of Pakistan (northeastern Arabian Sea) as an ideal archive for investigating the entire SAM system (Fig. 1). This core preserves finely laminated sediments during the GI-1 and benefits from a unique setting sensitive to both winter and summer monsoons. Arabian Sea ocean circulation and sedimentary deposition are predominantly controlled by the southeast SASM winds and associated summer season precipitation^7^. During the boreal winter, cold and dry northeasterly monsoon winds increase latent heat loss and evaporative cooling at the sea surface, determining the seasonally integrated sea surface temperature (SST) of the northeast Arabian Sea. As a result, an increase in SAWM winds leads to a lower annual mean SST^8–10^.

**Extended Data Fig. 1:**
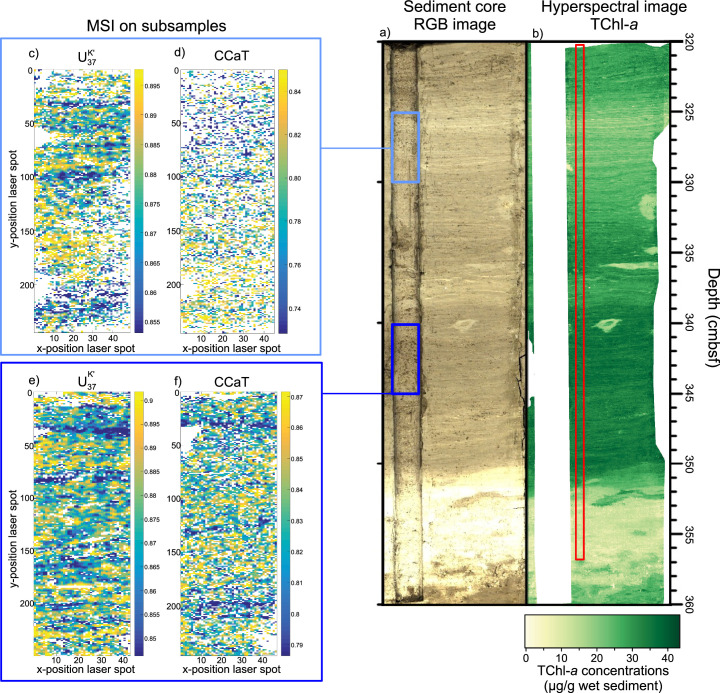
An illustrative example of mass spectrometry and hyperspectral imaging applied to sediment core SO130-289KL. **a**) RGB image of the sediment core SO130-289KL section from 3.20 to 3.60 m. **b**) Hyperspectral imaging of total chlorophyll-*a* and its colourful degradation products (TChl-*a*) concentrations on selected sediment section. The red rectangle indicates the area from which part of the data from this section used in this study were selected. Major disturbances were avoided, and the interval was chosen to be close to the mass spectrometry data. White areas indicate masked intervals where the sediment was heavily disturbed. **c**) Mass spectrometry imaging (MSI) of the subsample from 325–330 cm showing the spatial distribution of the ${{\rm{U}}}_{37}^{{\rm{K}}{\prime} }$ palaeothermometer. **d**) MSI of the subsample from 325–330 cm showing the spatial distribution of the crenarchaeol–caldarchaeol tetraether index (CCaT). **e**) MSI of the subsample from 340–345 cm showing the spatial distribution of the ${{\rm{U}}}_{37}^{{\rm{K}}{\prime} }$. **f**) MSI of the subsample from 340–345 cm showing the spatial distribution of CCaT. Note that in panels **c–f**, each datapoint on the heat map represents an individual measurement.

To address the resolution constraints inherent in traditional analytical methods, we employed advanced imaging techniques^11,12^ on sediment core SO130-289KL (Extended Data Fig. 1) to overcome sample size limitations of conventional techniques and capture micrometre-scale spatial resolution, that is, high-frequency temporal signals. Employing mass spectrometry imaging (MSI), we reconstructed SST variability relying on two biomarker-based SST proxies. We utilized the ${{\rm{U}}}_{37}^{{\rm{K}}{\prime} }$ alkenone unsaturation index as a reliable well-calibrated SST proxy in the region^13^. By contrast, a previously published study on a proximal core also utilizing MSI demonstrated that the cyclization of glycerol dialkyl glycerol tetraether (GDGT)-based crenarchaeol–caldarchaeol tetraether index (CCaT) is controlled by water column oxygenation, rather than SST variability^14^. In addition, hyperspectral imaging within the near-visible infrared range was conducted to elucidate chloropigments deposited in the sediment, serving as an indicator of marine primary production^12^. To further support our high-resolution data with information on hydroclimate and vegetation change, we performed conventional low-resolution carbon and hydrogen leaf wax isotope analyses.

## Seasonal SAM wind dynamics during the last deglaciation

**Fig. 2:**
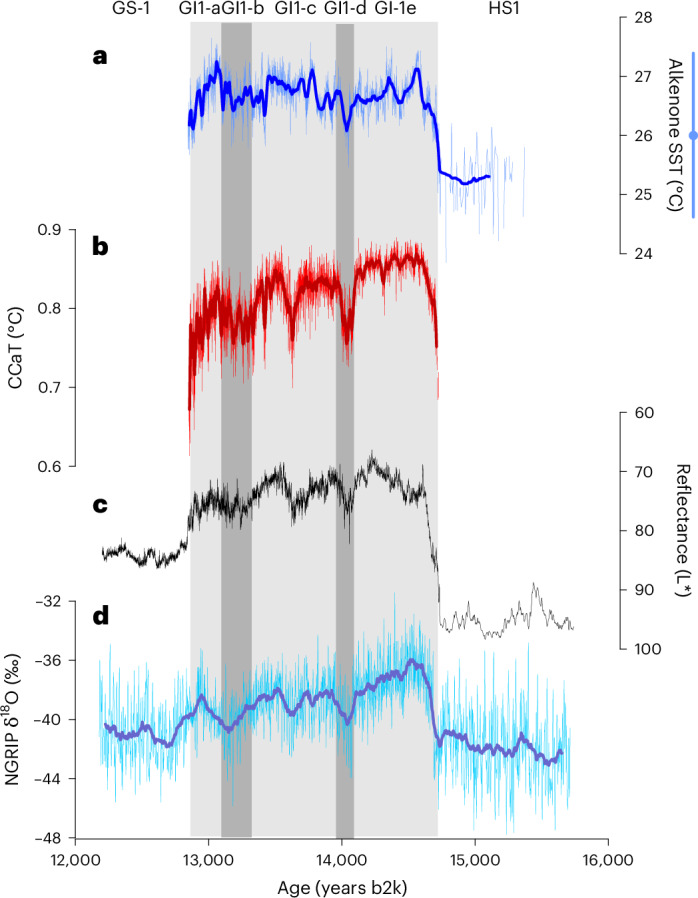
Proxy records from sediment core SO130-289KL and comparison with the Greenland ice core. **a**, MSI-derived alkenone-based SST (thick blue line represents a 50-data-point running average); the range of associated calibration uncertainty (±1.4 °C) is shown as error bars. **b**, MSI-based CCaT time series (thick red line represents a 50-data-point running average). **c**, Sediment reflectance^44^. **d**, Greenland NGRIP δ^18^O ice-core record^19^.

SST reconstruction during the GI-1 relied on the alkenone-based palaeothermometer obtained by MSI, allowing a spatial resolution of 200 µm, which translates into a temporal resolution of less than 1 year (Fig. 2). However, limitations of the age model do not allow reliable reconstruction of the variability at the resolution below the multidecadal time band. Detection of useful lipid biomarker signals via MSI was limited in bioturbated sediments deposited during Heinrich Stadial 1 (HS1) and GS-1 (Methods), and we thus consider only SST temporal variability during the GI-1, where the sediment is finely laminated. Alkenone-based SST indicates a prominent, abrupt increase of up to ~2 °C in just a few decades during the HS1 and GI-1 transition and reached values of 27.5 ± 1.4 °C. The SSTs during the GI-1 interstadial remained high between 26 ± 1.4 °C and 27.5 ± 1.4 °C, which is comparable to the present-day regional SST of 26.4 °C (ref. ^15^).

**Fig. 3:**
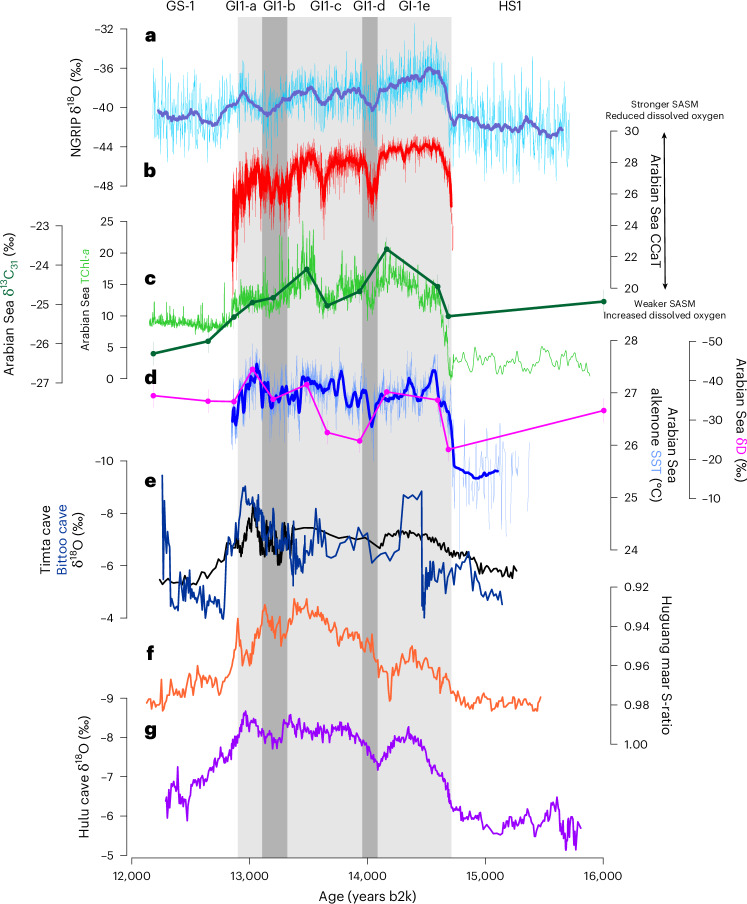
Comparison of various palaeoclimate records. **a**, Greenland NGRIP δ^18^O ice-core record^19^. **b**, Arabian Sea CCaT time series (thick red line is 50-data point running average). **c**, Arabian Sea TChl-*a* concentrations (light green) and δ^13^C values of *n*-C_31_ alkanes (thick dark-green line; error bars indicate analytical error of ±0.3‰). **d**, Arabian Sea alkenone-based SST (thick blue line is 50-data point running average) and precipitation δD (purple line; error bars indicate analytical error of ±3‰). **e**, Stalagmite δ^18^O records from Bittoo^46^ (blue line) and Timta^47^ (black line) caves. **f**, Huguang Maar S-ratio^22^. **g**, Hulu Cave δ^18^O^28^.

**Extended Data Fig. 2:**
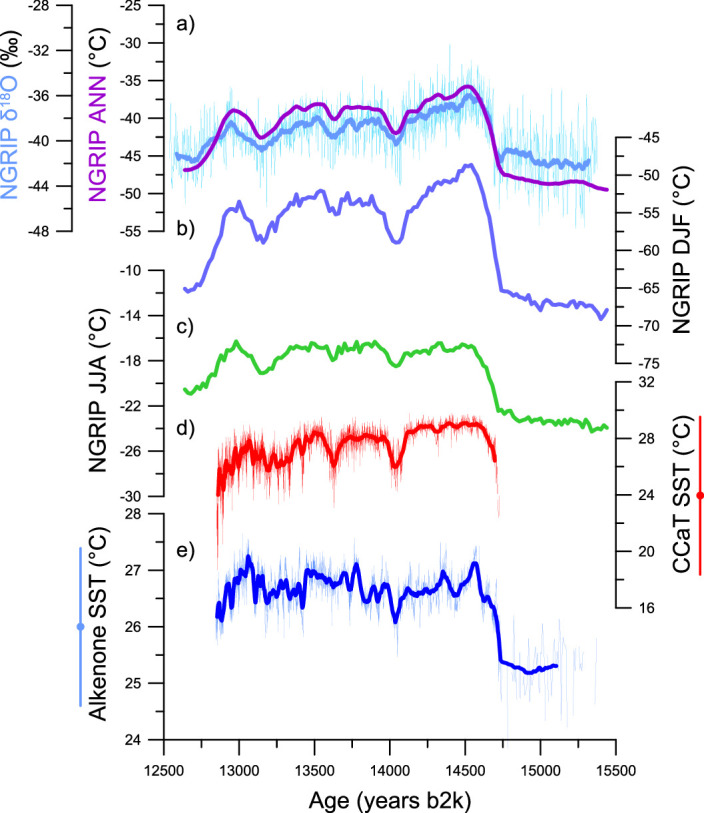
Comparison of Arabian Sea SST reconstructions with Greenland NGRIP temperature variability. **a**) Greenland NGRIP δ^18^O record^19^ (blue line; dark blue shows a 50-point running mean) alongside a data–model merged reconstruction of annual mean temperatures relative to present^81^ (purple line). **b**) Data–model merged reconstruction of summer (June–August; JJA) temperatures relative to present from the NGRIP ice core^81^. **c**) Data–model merged reconstruction of winter (December–February; DJF) temperatures relative to present from the NGRIP ice core^81^. **d**) MSI-derived alkenone-based Arabian Sea SST reconstruction (thin blue line; thick blue line shows a 50-point running mean). The range of associated calibration uncertainty of ±1.4 °C is represented as blue error bar. **e**) MSI-derived CCaT-based Arabian Sea SST reconstruction (thin red line; thick red line shows a 50-point running mean). The range of associated calibration uncertainty of ±5.6 °C is represented as red error bar.

CCaT values also suggest an abrupt response to the onset and end of the GI-1; however, the CCaT-based SST record exhibits remarkable differences compared with the alkenone-based SST record (Extended Data Fig. 2). Recent high-resolution SST reconstructions reveal that alkenone- and GDGT-based proxies capture distinct signals, where alkenone-based SST estimates are a more robust indicator of the SST variability^14,16,17^. Napier et al.^14^ demonstrated that seasonally resolved alkenone-based SSTs in the northeastern Arabian Sea align with instrumental SSTs on annual to decadal scales. By contrast, GDGT cyclization is proposed to respond to upwelling in the western Arabian Sea, where SASM-driven intensification of the clockwise ocean circulation pattern in the Arabian Sea reduces the dissolved oxygen concentration and enhances the intensity of the oxygen minimum zone (OMZ) in the northeastern Arabian Sea. A decrease in oxygen promotes GDGT cyclization^18^ and in the region of core location overpowers the SST signal (Supplementary Text 1). Therefore, we do not consider CCaT a reliable SST proxy, as it is to a large extent indicative of oxygen availability and OMZ intensity controlled by southeasterly summer winds intensity, serving as an indirect proxy for the SASM strength (Fig. 3).

The reconstructed deglacial CCaT time series closely aligns with Greenland ice-core δ^18^O data^19^ (Fig. 2), particularly with summer temperatures (Extended Data Fig. 2), strongly suggesting a North Atlantic and high-latitude Northern Hemisphere control on SASM, most likely by determining the position of the ITCZ^20^. Unlike CCaT, alkenone-based SST exhibit a less clear link to Greenland ice-core δ^18^O patterns, where only some prominent events appear in the alkenone-based SST time series, such as the distinct onset of the GI-1, cooling during the GI-1d and cooling at the transition into the GS-1 (Fig. 2). This suggests that changes in Northern Hemisphere high latitudes and Atlantic meridional overturning circulation had an influence on northeastern Arabian Sea SST but were not the primary driver of longer-term trends. Instead, alkenone data suggest a progressive SST increase, which is consistent with the Northern Hemisphere low- to mid-latitude progressive warming during the GI-1^4,21^. Alkenone-based SST indicates a warm early GI-1e (that is, weak SAWM) and cooling during the late GI-1e (that is, stronger SAWM), a pronounced SST drop in the GI-1d and low SST in the early GI-1c, with SST estimates generally under 27 ± 1.4 °C. This cooling phase was followed by a general trend of progressively increasing alkenone-based SST until the transition into the GS-1, albeit interrupted during the GI-1b. This pattern suggests a gradual weakening of winter monsoon winds in the late stage of GI-1, a trend that has also been inferred for the East Asian winter monsoon system based on the magnetic and elemental data from the Huguang Maar^22^ (Fig. 3), suggesting a coherent evolution of the entire Asian winter monsoon. However, we note that the use of Huguang Maar sediments as a winter monsoon proxy remains debated^23^, and proposed implications for large-scale Asian winter monsoon coupling should be interpreted with caution.

**Fig. 4:**
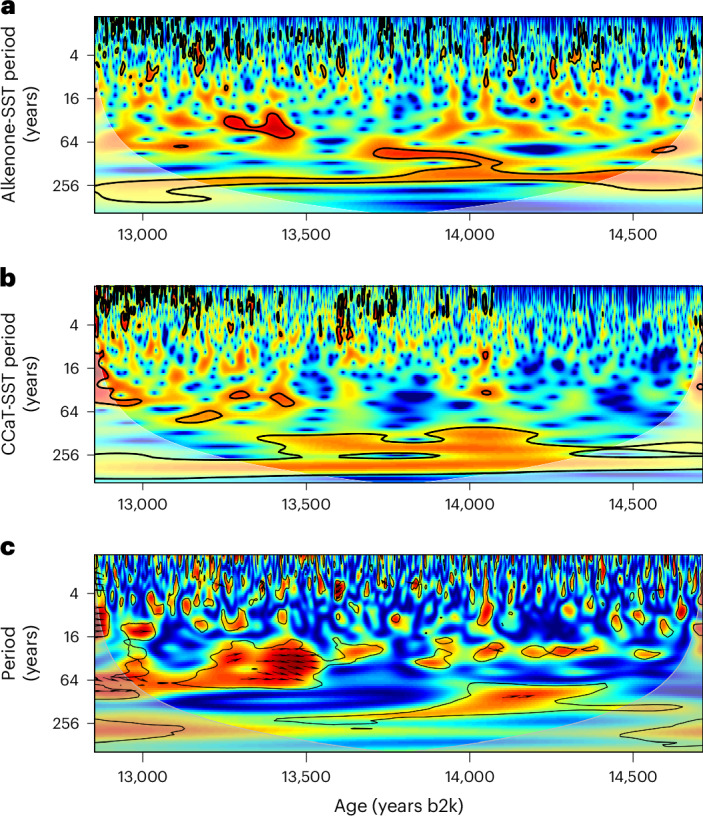
Periodicity and phase relation of alkenone- and CCaT-derived SSTs. **a**, Wavelet of alkenone-based SST (detrended for >500-year cycles). **b**, Wavelet of CCaT-based SST (detrended for >500-year cycles). Statistically significant periodicity intervals are highlighted. **c**, Cross-wavelet transfor (CWT) between the alkenone- and CCaT-based SSTs; it shows the coherence between the two SST records across different frequencies over time. Statistically significant regions of coherence are highlighted. In these areas, arrows indicate the phase relationships between the SST proxies: left-pointing arrows signify antiphase behaviour, while right-pointing arrows indicate in-phase behaviour.

**Extended Data Fig. 3:**
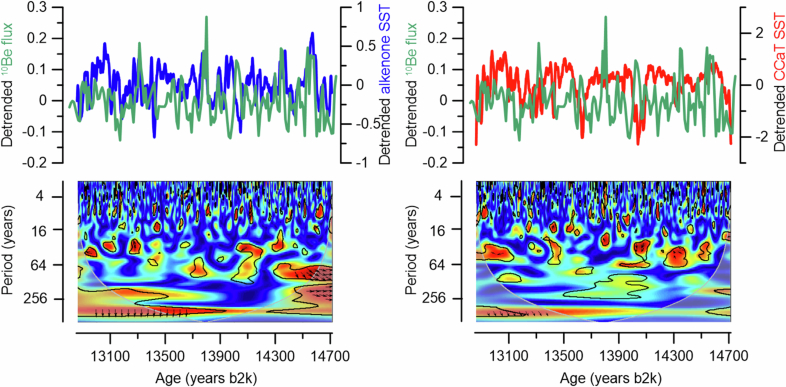
Cross-wavelet analyses between biomarker-based SSTs and ^10^Be flux^25^. On the left, comparison between alkenone-based SST and ^10^Be flux, while on the right CCaT-based SST comparison with ^10^Be flux. All time series were low-filtered (detrended) for periods longer than 500 years. SST time series are shown as a running average of 20 data points.

To assess the periodicity of alkenone-based SST and CCaT, we conducted wavelet analyses (Fig. 4). Neither time series shows consistent significant periodicity in decadal to multidecadal bands. Cross-wavelet analyses between these two time series do not show a consistent significant coherence in lower than centennial bands, supporting different drivers of these two proxies. Exceptions are GI-1a and GI-1b, where records show a significant in-phase relationship on multidecadal timescales. However, the lack of persistent periodicities in this interval more likely suggests an increased thermal influence on the CCaT proxy on multidecadal timescales, rather than a sustained phase relationship between summer and winter monsoon strength on these timescales, albeit we cannot rule out any of these scenarios. On multicentennial timescales, alkenone-based SST displays a strong 200–250-year cycle during the entire GI-1 (Fig. 4a), while CCaT shows similar periodicity only between 13,400 and 14,400 years b2k (Fig. 4b), suggesting a possible influence of the ~210-year de Vries solar cycle on monsoon variability^24^. However, cross-wavelet analysis reveals that, although both proxies show a 200–250-year periodicity, their coherence is not significant across the entire record, implying different feedbacks of the summer and winter monsoons to solar forcing (Fig. 4c). Therefore, we performed cross-wavelet analyses between Arabian Sea alkenone-based SST and CCaT with the ^10^Be flux from Greenland ice core^25^, as a record of past solar activity. Except for the interval between 13,900 and 14,300 years b2k, alkenone-based SST exhibits a significant in-phase relationship with the ^10^Be flux in the 200–250-year band (Extended Data Fig. 3). This pattern suggests solar forcing on alkenone-based SST, with warmer SST and a weaker SAWM occurring during periods of solar minima. By contrast, CCaT does not show as robust a phase relationship with the ^10^Be flux in the 200–250-year band as observed for alkenone-based SST. Nevertheless, it displays an out-of-phase link with the ^10^Be flux in both the 200–250-year and multidecadal bands (Extended Data Fig. 3), suggesting stronger SASM during periods of solar maxima, in agreement with previous studies^26^.

To better understand the SASM and SAWM evolution, we reconstructed total chloropigments-*a* (TChl-*a*; defined as chlorophyll *a* and chlorophyll derivatives) variability, a marine primary production proxy^12^, via hyperspectral imaging at ~84-µm resolution (Fig. 3). Primary production in the northeastern Arabian Sea is affected by strong SASM southwesterly winds, while a secondary peak occurs in winter when moderate and dry SAWM northeasterly winds initiate a convective winter mixing that provides nutrients for enhanced biological productivity^9^. The TChl-*a* in our record ranges from near 0 to 25 µg g^−1^ during the last deglaciation, where the values above 10 µg g^−1^ are associated with the GI-1 warm stage, while HS1 and GS-1 exhibit values lower than 10 µg g^−1^, suggesting limited primary production during the stadial stages (Fig. 3). During the GI-1, TChl-*a* variability generally correlates well with Greenland ice-core δ^18^O data, indicating a stark link to the Northern Hemisphere high latitudes, suggesting dominant SASM influence on primary production (Fig. 3). However, intervals with the highest alkenone-based SSTs during early GS-1e and GI-1a exhibit generally decreased primary production, suggesting a notable influence of a weakened SAWM on primary production via reduced convective winter mixing of the water column.

## Summer and winter precipitation regimes

Our micrometre-scale imaging-based proxy records are indicative of the monsoonal wind strength. However, the direct connection between these wind systems and seasonal precipitation remains uncertain. To shed further light on this, stable hydrogen (δD) and carbon (δ^13^C) isotope data of the sedimentary C_31_ leaf wax *n*-alkane were analysed as proxies for the isotopic composition of rainfall and vegetation evolution, respectively. δD values (−43 ± 3‰ to −23 ± 3‰; Fig. 3) reflect Indus River catchment precipitation dynamics, as this catchment is the main source of leaf wax *n*-alkanes. We note that, besides precipitation amount, isotope-based proxies, such as δD and δ^18^O, can be strongly affected by changes in moisture source areas and seasonality of precipitation^27,28^. In the northwestern Indian subcontinent, winter rains are primarily driven by western disturbances associated with the subtropical westerly jet^29,30^, which today can account for up to one-third of annual rainfall in northern India^31^, with up to 50% in Karakorum and the western Himalaya^32^. Vegetation dynamics provide an independent constraint on these seasonal changes. δ^13^C values of plant-wax *n*-alkanes are systematically more depleted than bulk plant organic matter owing to biosynthetic fractionation^33^, and obtained values between −26.3 ± 0.3‰ and −23.6 ± 0.3‰ indicate a substantial contribution of C_4_ plants to the Indus River catchment vegetation. In many monsoon-dominated areas, higher C_4_ plant abundance is driven by pronounced rainfall seasonality with high summer precipitation and notable winter water stress, while a decrease in seasonality of precipitation favours C_3_ plants^34–36^.

**Extended Data Fig. 4:**
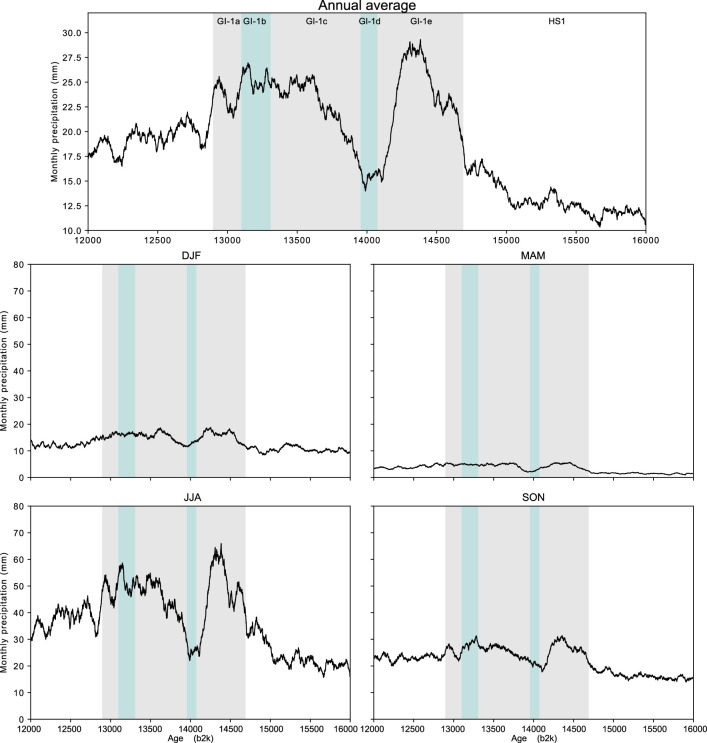
TraCE-21ka annual and seasonal precipitation simulations^37,82^. Top: Annual precipitation simulation. Middle: winter (December, January, February; DJF) and spring (March, April, May; MAM) precipitation simulations. Down: summer (June, July, August; JJA) and autumn (September, October, November; SNO) precipitation simulations.

**Extended Data Fig. 5:**
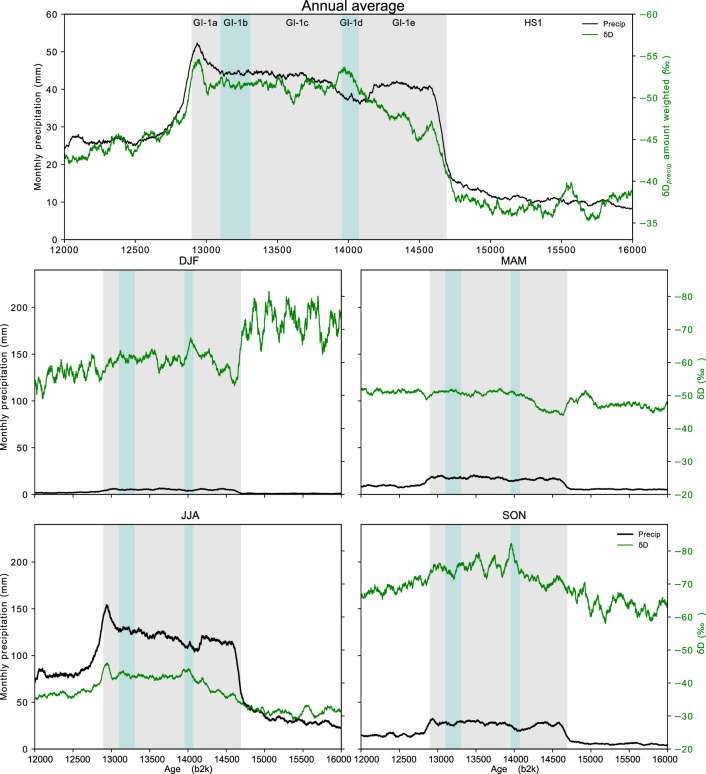
iTraCE- annual and seasonal precipitation (black lines) and δD of precipitation simulations (green lines)^38,73^. Top: annual precipitation and δD simulation. Middle: winter (DJF) and spring (MAM) precipitation and δD simulations. Down: summer (JJA) and autumn (SNO) precipitation and δD simulations.

The GI-1e period exhibits low δD values (Fig. 3), suggesting increased precipitation, which is associated with an increase in southwest SASM winds, as supported by CCaT and sediment reflectance (Fig. 2). The more positive δ^13^C values suggest high C_4_ plant abundance, implying high summer rainfall and strong winter water stress due to low winter precipitation, additionally supported by iTRACE and TraCE-21ka simulations^37,38^ (Extended Data Figs. 4 and 5). An increase in δD and a decrease in δ^13^C values during the GI-1d and the early stage of GI-1c suggest reduced summer precipitation and a subsequent decline in C_4_ plants due to a less pronounced seasonal precipitation contrast. However, we observe a regime shift from GI-1b, which is characterized by concurrent decreases in δ^13^C and δD (Fig. 3). Lower δD commonly reflects enhanced precipitation; nonetheless, we posit a precipitation increase during winter, as only an increase in winter precipitation can explain observed C_3_ plant expansion by reducing precipitation seasonality and winter water stress.

During GS-1, δ^13^C reaches its most negative values within the studied interval, implying a pronounced reduction in precipitation seasonality. While δD values increase relative to GI-1a, they remain comparable to or lower than those of GI-1e-c. This pattern suggests that, despite a weakening of the SASM during GS-1, annual precipitation isotopic signal remained comparable to most of GI-1. However, we emphasize that rainfall seasonality exerts a major effect on the isotope signal. Winter precipitation delivered by long-range westerly jets transport (for example, from the Mediterranean; Fig. 1b) undergoes progressive upstream rainout, resulting in isotopically depleted values compared with locally recycled or convective precipitation^39^. An increase in winter rainfall from those distant sources can thus disproportionately lower δD values. Consequently, intervals dominated by summer monsoon precipitation, such as GI-1e-c, may exhibit greater total precipitation than intervals with enhanced winter rainfall, such as GS-1, but similar mean δD. This is supported by the iTRACE simulation^38^, which suggests that winter precipitation δD values were lower than summer δD values throughout the deglaciation (Extended Data Fig. 5).

We posit that, akin to modern conditions, the rise in global temperatures during the deglaciation^21^ led to the progressive intensification of the subtropical westerly jets and western disturbances contribution to the Hindu Kush, Karakoram and western Himalaya in the winter months by increasing the frequency and lengthening the season of western disturbances^40^. Moreover, the precession forcing during the last deglaciation led to progressively increasing extreme seasonal contrasts, with decreasing winter and increasing summer insolation. This intensifying seasonality progressively steepened upper-tropospheric temperature gradients and, via thermal wind balance, strengthened the subtropical westerly jet during the extended winter and spring seasons, while also enhancing a southerly displaced Mediterranean winter storm track and associated westerlies-driven precipitation^41^. The progressively increasing influx of isotopically depleted Mediterranean-sourced winter moisture during the last deglaciation led to more negative δD values in precipitation and plant waxes. We further observe that the progressive increase in winter precipitation coincides with a weakening of the monsoon winds (Supplementary Text 2), suggesting that both hydroclimatic components were potentially modulated by a common large-scale forcing mechanism.

## Implication of the SASM and SAWM variability

The Asian monsoon system is primarily driven by orbital forcing^28,42,43^ but strongly modulated through changes in the strength of the Atlantic meridional overturning circulation and its influence on ITCZ position on millennial timescales^44,45^. Greenland interstadials (stadials) generally align with stronger (weaker) summer monsoon, as the ITCZ moves towards the warming hemisphere^2^. However, during the GI-1, speleothem δ^18^O data, as some of the best-resolved and most precisely dated palaeoclimate records of the Asian monsoon on these timescales, diverge notably from Greenland ice-core data (Fig. 3). Speleothem δ^18^O data progressively decrease towards the end of the interstadial^5,28,46,47^ alongside general trends of Northern Hemisphere high-latitude cooling (Fig. 3). By contrast, our findings strongly suggest that the SASM winds were primarily controlled by Northern Hemisphere high-latitude climate. We posit that the stark decrease in speleothem δ^18^O values in South Asia during the GI-1a–c (Fig. 3e) is not driven solely by an increase in summer precipitation, but instead, similarly to δD in our record, reflects an isotopic signal biased to the increased winter precipitation from distant sources during the GI-1a-c, driven by intensified westerly jets and western disturbances with a more negative isotopic signature. Moreover, we emphasize that the effect of temperature on monsoon rainfall is low; however, temperature plays an important role in controlling the isotopic composition of precipitation associated with westerly jets. Thus, an increase in the proportion of winter precipitation additionally lowers the mean annual δ^18^O values through the temperature effect^41^.

**Extended Data Fig. 6:**
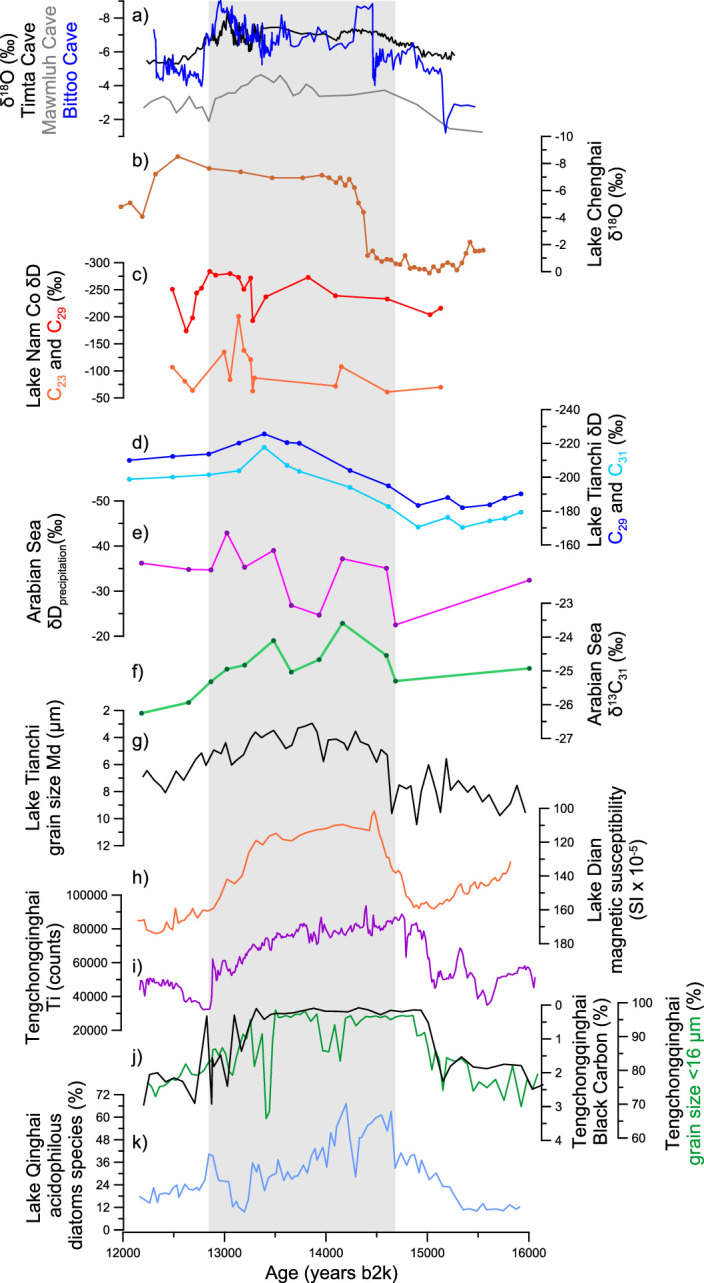
Isotope and non-isotope proxies indicative of South Asian monsoon precipitation. **a**) Stalagmite δ^18^O records from Mawmluh^49^ (brown line), Bittoo^50^ (blue line) and Timta^52^ (black line) caves. **b**) Lake Chenghai δ^18^O^48^. **c**) Lake Nam Co δD of *n*-C_23_ (orange line) and *n*-C_29_ (red lines) alkanes^49^. **d**) lake Tianchi δD of *n*-C_29_ (blue line) and *n*-C_31_ (light blue lines) alkanes^4^. Arabian Sea **e**) δD of precipitation and f) δ^13^C values of *n*-C_31_ alkanes. **g**) lake Tianchi median grain size^4^, **h**) Lake Dian magnetic susceptibility^52^. Tengchongqinghai^50^ i) Ti and **j**) black carbon (black line) and grain size <16 µm (green line). **k**) Lake Qinghai acidophilous diatoms^51^.

Climate models yield conflicting results regarding the plausibility of the proposed seasonal bias in the proxy record (Supplementary Text 3). Therefore, we examined additional palaeoclimate proxy records reflecting SAM dynamics during deglaciation. Lacustrine δ^18^O and δD records from SAM-dominated regions show decreasing values towards the end of the GI-1^4,48,49^, consistent with speleothem δ^18^O and Arabian Sea δD records (Extended Data Fig. 6a–e). By contrast, non-isotopic multiproxy lake records reflecting lake levels, relative humidity and precipitation^4,50–52^ do not support a progressive precipitation increase during the GI-1c to GI-1a (Extended Data Fig. 6f–k). This suggests that the GI-1a-c and GS-1 lower isotope values are related to a change in rainfall seasonality and moisture source area, rather than a substantial increase in summer monsoon precipitation. While there are broad similarities between our δD and other Asian monsoon records, the seasonal bias mechanism proposed here cannot be directly applied to the entire Asian Monsoon system, and further research is needed to assess the role of large-scale atmospheric forcing on winter precipitation across these regions.

In summary, our data strongly suggest that the SASM responded directly to changes in high-latitude Northern Hemisphere climate during the last deglaciation. Meanwhile, alkenone-based SST suggests that the northeastern Arabian Sea SST and SAWM responded promptly to deglacial abrupt climate transitions, but that the general trends within the GI-1 were influenced by additional mechanisms. Our findings highlight a previously unrecognized mechanism controlling the winter monsoon winds and non-monsoonal precipitation dynamics, where the deglacial rise in global temperature with orbital forcing movement towards the precession minimum affected a long-term increase in boreal winter precipitation and gradual weakening of the SAWM strength.

## Methods

### Study site and chronology

Sediment core SO130-289KL (23°07.34′ N, 66°29.84′ E; 571 m water depth) was collected from the Sindh continental margin within the present-day OMZ, which spans approximately 200 to 1,200 m water depth^54^. This 20.2-m piston core was retrieved from a levee along the flank of the Indus submarine canyon near the Indus River mouth.

The age model has been previously established by ref. ^44^. In short, the first-order age model for the entire 20.2-m SO130-289KL core is based on 20 accelerator mass spectrometry ^14^C dates. The age model is further fine-tuned by correlating the high-resolution total colour reflectance (L*) records to the ice-core δ^18^O record of North Greenland Ice Core Project (NGRIP) using the GICC05 age scale^55,56^, showing a remarkable consistency with the Cariaco Basin L* record^44^. Ultimately, the interval between HS1 to GS-1 was constrained by six tie points^57^. Because of this fine-tuning, it is not possible to interpret lead or lag relationships between the NGRIP and our proxy records.

### MSI

MSI was performed on sediment deposited between 290 and 355 cm below the seafloor. The core was subsampled with one 30-cm-long and two 20-cm-long LL channels, which were subsequently X-rayed to produce photographs with clearly marked 5-cm intervals. Each sample was divided into 5-cm-long subsamples, freeze-dried and stabilized by embedding in a mixture of 5% gelatin and 1% sodium carboxymethyl cellulose before freezing^58,59^. Subsamples were then sectioned using a cryomicrotome into slices of either 60 µm (alkenone analysis) or 100 µm (GDGT analysis). The slices were sequentially obtained, ensuring comparability of data for both proxies with minimal differences in the sedimentary matrix. The slices were mounted on indium tin oxide-coated glass slides and dried in a desiccator.

MSI was performed using a 7 T solariX XR Fourier-transform ion cyclotron resonance mass spectrometer equipped with a matrix-assisted laser desorption/ionization source and a Smartbeam II laser (Bruker Daltonik). Analyses were conducted in positive ionization mode, and data acquisition was optimized for targeted biomarkers using the continuous accumulation of selected ions mode. For alkenones, the mass-to-charge ratio (*m*/*z*) range was set at 554 ± 12, while for GDGTs, it was 1,320 ± 10. A rastering approach with a 200-µm spot distance ensured spatial resolution, and data acquisition was performed with no data reduction for alkenones and 75% data reduction for GDGTs using ftmsControl 2.1.0 (Bruker Daltonik). Exported mass spectra included spot positions, *m*/*z*, intensity and signal-to-noise ratio (SNR). Subsequent data analysis employed MATLAB R2017a to identify compounds of interest with a mass accuracy of ±0.003 Da for alkenones and ±0.01 Da for GDGTs, with an SNR threshold of 5 for alkenones and a lower SNR threshold of 0.5 for GDGTs due to the nearly noise-free nature of the data following data reduction.

To correct for potential expansion of the sedimentary matrix during preparation, the dimensions of the mapped areas were aligned with the original X-ray images. Tie points were identified visually in both datasets following procedures outlined in refs. ^16,58^. Using fitgeotrans in MATLAB, molecular proxy data were mapped onto the X-ray images. For alkenones, the intensity of C_37:2_ and C_37:3_ species relevant to the proxy was recorded for each laser spot. ${{\rm{U}}}_{37}^{{\rm{K}}{\prime} }$ was calculated following equation (1).

1 $${{\rm{U}}}_{37}^{{\rm{K}}{\prime} }={{\rm{C}}}_{37:2}/\left({{\rm{C}}}_{37:2}+{{\rm{C}}}_{37:3}\right).$$

MSI-based analyses cannot distinguish between all components that are included in the conventionally used GDGT-based SST proxy, such as TEX_86_, because *m*/*z* values of crenarchaeol and its isomer, which is included in the proxy, are identical. Therefore, here we focus on an accessible ratio of the two major core lipids, GDGT-0 (caldarchaeol) and GDGT-5 (crenarchaeol and its isomer), in the form of the CCaT^14,16,17^. Only spots where both target compounds were detected were considered for subsequent analysis, with intensity values summed over depths corresponding to 200-µm layers. Data quality criteria were satisfied when at least ten spots containing both compounds were available per horizon^60^. CCaT was calculated following equation (2).

2 $$\mathrm{CCaT}=\left(\mathrm{GDGT}5+\mathrm{GDGT}5{\rm{\mbox{'}}}\right)/\left(\mathrm{GDGT}0+\mathrm{GDGT}5+\mathrm{GDGT}5{\rm{\mbox{'}}}\right).$$

GDGTs were below the detection limit in bioturbated sediments of HS1. Alkenone detection in this interval was challenging due to low concentrations; however, it allowed the generation of a partial ${{\rm{U}}}_{37}^{{\rm{K}}{\prime} }$ record that can be used to estimate SST in this interval, but is not well suited for studying variability in the time series. Bioturbated sediment characterizing the GS-1 was not studied by MSI.

The MSI-based ${{\rm{U}}}_{37}^{{\rm{K}}{\prime} }$ values were consistently lower than the ${{\rm{U}}}_{37}^{{\rm{K}}{\prime} }$ values derived from gas chromatography–flame ionization detection (GC–FID) measurements on the 2-cm-size samples from the same depth (equation (2)). This was already demonstrated by refs. ^14,57^ in the low latitudes with high SST, where MSI underestimates the GC–FID-based values. Therefore, we adjusted the MSI-${{\rm{U}}}_{37}^{{\rm{K}}{\prime} }$ values, where we summed MSI-based values to 2-cm intervals and compared them with extraction-based GC–FID estimates from the same depth intervals (Supplementary Fig. 1):

3 $$\mathrm{MSI}-{{\rm{U}}}_{37}^{{\rm{K}}{\prime} }={0.7603}^{* }\mathrm{GC}-\mathrm{FID}-{{\rm{U}}}_{37}^{{\rm{K}}{\prime} }+0.2658.$$

The alkenone unsaturation index was converted to temperature according to global calibration by ref. ^13^:

4 $$\mathrm{SST}\left(^\circ {\rm{C}}\right)=-0.957+54.3\left({{\rm{U}}}_{37}^{{\rm{K}}{\prime} }\right)-52.9\left({{\rm{U}}}_{37}^{{\rm{K}}{\prime} }\right)2+28.3\left({{\rm{U}}}_{37}^{{\rm{K}}{\prime} }\right)3.$$

We applied the nonlinear SST calibration from ref. ^13^ as it is based on alkenones from surface water particulates collected from the world ocean (that is, the natural environment of the alkenone producers) and relates to the temperatures measured at those sampling depths. The reduction in slope for water temperatures >24 °C is in line with the alkenone temperature calibration from ref. ^61^ based on surface sediment samples collected in the Indian Ocean.

To assess the uncertainty of SST estimates, we considered the analytical uncertainty related to the conversion of MSI data to quasi-gas chromatography data, which historically serve as the basis for calibration to SST. This uncertainty of ±0.009 units is defined by the standard error of the linear regression between the 2-cm integrated values of MSI-based analyses and related GC–FID-based estimates, and translates to approximately ±0.3 °C. On top of this range, the uncertainty of the global calibration from ref. ^13^ of ±1.1 °C was applied, resulting in a range of ±1.4 °C.

For this study, to calibrate CCaT to SST, we relied solely on the calibration presented by ref. ^14^. The calibration equation used is

5 $$\mathrm{SST}\left(^\circ {\rm{C}}\right)=-21.916\left(\frac{1}{\mathrm{CCaT}}\right)+54.428.$$

This calibration is associated with an uncertainty of ±5.6 °C, as reported by ref. ^14^. We did not apply a correction factor to the MSI-CCaT values compared with liquid chromatography (LC) data, as Napier et al. ^14^ demonstrated that such a correction is unnecessary due to negligible offsets between MSI-CCaT and LC-CCaT values measured on the same samples.

### Conventional alkenone analyses

Samples were extracted with a Dionex ASE 200 in three cycles using dichloromethane as the eluent. Extracts were dried under a stream of nitrogen, saponified with 0.5 ml 1-propanolic KOH (5%) for 24 h at 20 °C, followed by a solid phase clean-up using silica gel columns to remove the KOH. These purified extracts were analysed by gas chromatography with an HP-6890 instrument equipped with an HP PTV Inlet on a DB-1 capillary column (30 m × 0,25 mm inner diameter; film thickness 0.25 µm) coupled to a flame ionization detector. Samples were injected splitless in dichloromethane using a cool injection program with solvent venting. Hydrogen was the carrier gas at a flow of 0.9 ml min^−1^. A temperature program of 2 min isothermal at 56 °C, 56–150 °C at 24 °C min^−1^, 150–320 °C at 4.7 °C min^−1^ and 10 min isothermal was used, giving a good separation of all major compounds. Alkenones were identified by retention times.

### Hyperspectral imaging

Hyperspectral imaging was carried out using a Specim PFD-CL-65V10E linescan camera following the methods from ref. ^62^ with a resolution of 84 × 84 μm (pixel size). The data were processed using ENVI software^62^. Relative absorption band depth (RABD) indices were calculated to quantify absorption features associated with sedimentary pigments. The RABD671 index represents total chlorophyll (TChl: mainly chlorophyll *a*, *b* and coloured transformation products) and is interpreted as representative of total algal abundance^63^. The index was normalized for the matrix signal by division RABD671 with mean reflectance (Rmean). The matrix-normalized index was calibrated to concentrations (μg g^−1^ wet sediments) via linear regression between measurements of extracted sedimentary pigments and RABD671/Rmean values of the same sample depths on 13 samples and 3 full chemical replicates and 8 analytical replicates (24 total measurements) from a 4-m-long sediment section covering the interval between the Holocene and HS1. The proxy–proxy calibration was done using an ordinary least-squares regression (*n* = 16, *R*^2^ = 0.62, root mean square error of prediction 24%).

Sedimentary pigments were extracted using the combined protocols from refs. ^64,65^. Pigments from 1 g of wet sediment were extracted by 2 ml of 100% acetone (high-performance liquid chromatography grade), sonication and maceration over 12 h at −20 °C until the supernatant was pale in colour. After a succession of acetone extraction steps, the supernatant with extracted pigments was decanted into a 20-ml amber glass vial and stored in the dark at −20 °C. The concentration of TChl in the extracts was analysed using a Shimadzu ultraviolet–visible spectrometer (UV-1800 CE 230 V; 300–800 nm) at a 0.1-nm step size spectral resolution. The concentration of total green pigments (for example, chlorophyll *a* and their degradational products such as pheophytins and pheophorbides) was determined based on the absorbance at 666 nm using the Lambert–Beer law and the specific extinction coefficient in an acetone:water (90:10) solution^66^. The presented total green pigments are expressed in μg g^−1^ of wet sediment using the sample weight and the final volume of the extract.

### Hydrogen and carbon stable isotopes

Total lipid extracts from the samples were split into lipid classes of different polarity by elution over a 4-cm, deactivated Si-column: apolar fractions were obtained by elution with hexane and polar fractions by elution with dichloromethane/methanol = 1/1. The apolar fractions were further cleaned by elution over AgNO_3_-coated Si-columns with hexane to remove unsaturated compounds. Long-chain *n*-alkanes in the samples were quantified on a Focus GC–FID by comparing peak areas with those of external alkane mixtures.

δ^13^C analyses of the *n*-C_31_ alkane were conducted using a Thermo Fisher Scientific MAT 252 isotope ratio mass spectrometer coupled, via a GC-C combustion interface with a nickel catalyst operated at 1,000 °C, to a Thermo Scientific Trace GC equipped with an HP-5ms column (30 m × 0.25 mm × 0.25 μm film thickness). Each sample was measured at least in duplicate. δ^13^C values were calibrated against a CO_2_ reference gas of known isotopic composition and are reported in ‰ relative to VPDB (Vienna Pee Dee Belemnite). Accuracy and precision were determined by measuring *n*-alkane standards calibrated against the A4-Mix every six measurements. For δ^13^C, the difference between the long-term means and the measured standard values yielded a 1*σ* (one standard deviation) error of 0.3‰. Precision and accuracy of the squalane internal standard were 0.1‰ and 0.4‰, respectively. Precision of the replicate analyses of the *n*-C_31_ alkane was 0.1‰ on average.

δD analyses of the *n*- C_31_ alkane were conducted on a Thermo Scientific MAT 253 Isotope Ratio Mass Spectrometer coupled via a GC IsoLink operated at 1,420 °C to a Thermo Fisher Scientific TRACE GC equipped with a HP-5ms column (30 m × 0.25 mm × 0.5 μm). δD values were calibrated against a H_2_ reference gas of known isotopic composition and are given in ‰ VSMOW (Vienna Standard Mean Ocean Water). Each sample was measured at least in duplicate if sufficient material was available. Accuracy and precision were controlled by a laboratory internal *n*-alkane standard calibrated against the A4-alkane mixture (provided by A. Schimmelmann, Indiana University) analysed every sixth measurement, and through daily determination of the H_3_^+^ factor. Measurement precision was determined by calculating the difference between the analysed values of each standard measurement and the long-term mean of standard measurements, which yielded a 1*σ* error of 3‰. Daily H_3_^+^ factors were 5.13 ± 0.03. Precision and accuracy of the squalane internal standard were both 1‰. Precision of the replicate analyses of the *n*-C_31_ alkane was 1‰ on average. For samples that could only be analysed once and for the analytical replicates with better precision, the long-term precision of the standards (3‰) was assumed. Different hydrogen isotope fractionation factors of different vegetation types can cause offsets in δD_31_. Therefore, to more reliably estimate δD of precipitation, we used δ^13^C values as an estimate for vegetation type, according to established procedures in refs. ^67,68^. Finally, δD of precipitation estimates were corrected for global ice-volume changes affecting isotopic compositions in the hydrological cycle. For this correction, Bintanja’s marine oxygen isotope compilation^69^ was used, assuming a global δ^18^O change of 1‰ VSMOW from the Last Glacial Maximum to the present^68^.

### Wavelet and CWT analyses

Wavelet transform analyses we performed in R Statistic using astrochron^70^ and biwavelet libraries. Data were previously detrended for periodicity longer than 500 years. Statistically significant regions of periodicity are highlighted. We calculated the phase relationship between the alkenone and CCT-based SST time series using cross-wavelet transform (CWT) analysis, as implemented in the wtc function, which is part of the biwavelet package for R (refs. ^17,71,72^). The CWT shows the coherence between the two SST records across different frequencies over time. The CWT between the SST time series and ^10^Be flux was calculated in the same way on the detrended time series for periods longer than 500 years. Statistically significant regions of coherence are highlighted. In these areas, arrows indicate phase relationships between the SST proxies. Left-pointing arrows signify antiphase behaviour, whereas right-pointing arrows indicate in-phase behaviour.

### TraCE-21ka and iTRACE simulations

The TRACE simulation was performed with the CCSM3 model developed by the National Center for Atmospheric Research, and is a full complexity-coupled atmosphere-ocean model. For the transient palaeoclimate runs, CCSM3 was forced with changes in greenhouse gas concentrations, orbital insolation, continental ice sheets and the resulting sea level changes, as well as fresh water forcing from melting ice sheets and drainage of proglacial lakes^37^. The TraCE-21ka spatial resolution for the atmosphere was T31_gx3, (3.75° × 3.75°, ∼415 km).

In addition, the state-of-the-art isotopic-enabled transient climate model simulation iTRACE for 20–11 ka BP was analysed^38,73^. iTRACE was run using iCESM, largely following the modelling strategy of TraCE-21ka, but additionally simulates precipitation δD and δ^18^O, and was run at a higher model resolution (1.9° × 2.5°). The δD in the model was filtered to exclude months with precipitation <1 mm, due to the very negative simulated δD values during those months. The annual δD was amount weighted as (δD_precip_month_ × Precip_month_)/*P*_annual_, where δ_Dprecip_month_ is the monthly precipitation δD value, Precip_month_ is the monthly precipitation amount, and P_annual_ is the total annual precipitation amount. The isolated effect of each forcing (ice-sheet configuration and sea level, orbital forcing, greenhouse gases and meltwater forcing) was derived as detailed in ref. ^73^. The time-series data from TraCE-21ka and iTRACE were derived from 70–75° E and 29–32° N, and presented as 100-year running means.

### Trajectory analyses

To analyse the moisture transport to the investigated location, we run a trajectory analysis using the Hybrid Single-Particle Lagrangian Integrated Trajectories (HYSPLIT) trajectory model of the Air Resources Laboratory of the National Oceanic and Atmospheric Administration^74^. Due to limited accessibility of meteorological data from Pakistan and India, we focused on the global observation data of the Climate Prediction Center via the KNMI Climate Explorer website. In detail, we investigated the precipitation in the catchment area of the Indus River from 70–75° E and 29–32° N between 1980 and 2024. This inland receptor location was chosen to capture an integrated signal of the entire lower–middle Indus catchment, reflecting continental moisture transport and terrestrial vegetation influences, while avoiding localized coastal or marine boundary-layer effects. For any day with precipitation of more than 1 mm per day over the whole area, we calculated the back trajectories expiring at the centre of the defined rectangle (72.5° E, 30.5° N) at 850 hPa (~1,500 m altitude), a standard level for monsoon wind analyses. Because we analysed a large modelled dataset rather than direct meteorological measurements, only values above 1 mm per day were used to avoid model-related uncertainties. To disentangle the different origins of the air masses, we calculated the trajectories for 240 h back in time. We performed the analysis for winter (December, January, February) and summer (June, July, August) to show the two different meteorological systems with different isotope compositions^75^.

## Online content

Any methods, additional references, Nature Portfolio reporting summaries, source data, extended data, supplementary information, acknowledgements, peer review information; details of author contributions and competing interests; and statements of data and code availability are available at 10.1038/s41561-026-02023-z.

## Supplementary information

Supplementary Information Supplementary Discussion (Supplementary Texts 1–3), Figs. 1–4 and References.

Peer Review File

## Data Availability

The published data are archived in the PANGAEA data repository and are available in refs. ^76–79^.

## Code availability

The code used for the back-trajectory calculations and for generating Fig. 1 is available via Zenodo at 10.5281/zenodo.20133814 (ref. ^80^).
