# Supplementary material for: Contrasting drivers of South Asian summer and winter monsoon evolution during the last deglaciation
Source: Nat Geosci. 2026 Jun 22;19(8):959–65. doi: 10.1038/s41561-026-02023-z (PMC13447086; doi:10.1038/s41561-026-02023-z)
Supplement: Supplementary file 1 [file 41561_2026_2023_MOESM1_ESM.pdf]

nature geoscience

Supplementary information

https://doi.org/10.1038/s41561-026-02023-z

Contrasting drivers of South Asian summer and winter monsoon evolution during the last deglaciation

In the format provided by the authors and unedited

Supplementary Information

Contrasting drivers of South Asian summer and winter monsoon evolution during the last deglaciation

Igor Obreht, Andreas Lückge, Mahyar Mohtadi, Petra Zahajská, Enno Schefuß, Denis Scholz, Lars Wörmer, Petter Hällberg, Alexander Budsky, Florian Adolphi, Gerald Haug, Martin Grosjean, Kai-Uwe Hinrichs

Supplementary Discussion

Supplementary Text 1

CCaT as a proxy for the South Asian summer monsoon

Our CCaT time series from the sediment core SO130-289KL exhibits a clearly different trend from $U_{37}^{K'}$ unstaturation index, which is considered to more reliably represent SST. This is in agreement with the observations from ref.[1], which applied mass spectrometry imaging and elemental mapping to reconstruct SST and precipitation intensity from 1900 AD using nearby box core SO90-58KG (24° 46.60' N, 65° 49.18' E, 876 m water depth) and compare it to instrumental records. They concluded that the alkenone-based SST reliably captures interannual variability, while seasonal variability seems to be muted. On the other hand, they observed that the CCaT proxy does not directly reconstruct sub-annual and interannual SST but instead reflects ocean current alternations linked to upwelling in the western Arabian Sea. Specifically, high CCaT values correspond to southeastward-moving currents originating from upwelled waters off the Oman coast, while low values align with northwestward-moving currents from the Bay of Bengal, strongly suggesting a water mass control on CCaT.

Consequently, authors from ref.[1] argued that the CCaT signal is indirectly influenced by the strength of western Arabian Sea upwelling, which is driven by the southwest SASM. Stronger upwelling leads to higher nutrient loads and oxygen depletion, which impacts the growth of Thaumarchaeota, key organisms in GDGT production. Lower oxygen availability results in increased GDGT cyclization, elevating CCaT values[2,3]. Conversely, reduced upwelling increases oxygen levels and lowers CCaT values. Authors also observed that CCaT exhibits a lagged (1–4 years) anti-phase relationship with the

Webster-Yang monsoon circulation index, reflecting the delayed response of ocean currents and oxygen cycling to monsoon intensity. Thus, CCaT at the location is suggested to serve as an indirect, potentially 1-4 years lagged proxy for western Arabian Sea upwelling, primarily driven by monsoon-related ocean current changes and oxygen availability[1].

These observations agree with our findings, since our CCaT reconstruction generally agrees with the main trends from sediment reflectance (Fig. 2), which reflects the combined signal of terrigenous and biogenic contributions influenced by precipitation and primary production, as well as OMZ intensity, all strongly SASM-related. However, this relationship does not allow to directly conclude if the CCaT was driven by the oxygen concentration[2,3] due to OMZ variability or nutrient contribution and primary production[4–6]. To additionally disentangle the influence of marine primary production and OMZ strength, we reconstructed total chloropigments-a (TChl-*a*) variability, a marine primary production proxy[7].

TChl-*a*, as an independent proxy for primary production exhibits very similar variability to L*. In contrast, differences on sub-centennial scales can be observed between CCaT and these proxies. This supports that CCaT was not driven by primary production, particularly on sub-centennial time scales. Alternative drivers of GDGT cyclization, such as nutrient concentration and ammonia oxidation rates appear secondary or negligible in this region during the deglaciation, given that high ammonia oxidation rates related to strong OMZ intensity would inversely impact CCaT-based SST values[6,8]. Considering high CCaT values during the GI-1 when compared to HS1 and to the GS-1, we also argue for an absence of major upper-ocean temperature reorganization as observed in the upwelling region of the western Arabian Sea[9]. Consequently, in agreement with conclusions from ref.[1], we consider CCaT at this site as an indirect proxy for western Arabian Sea upwelling, determined by monsoon-driven currents influencing the oxygen availability at the core location.

Supplementary Text 2

Long-term evolution of $\delta^{13}C$ and δD and implications for precipitation seasonality

In our record, both $\delta^{13}C$ and δD records exhibit a progressive long-term decrease from HS1 to 12,000 years b2k, upon which centennial-scale oscillations during GI-1 are superimposed. We argue that this gradual evolution indicates a sustained response to orbital-scale and deglacial boundary conditions

rather than solely short-lived monsoon variability. The long-term decline in δD is consistent with an increasing contribution of isotopically depleted Mediterranean-sourced winter moisture over the course of the deglaciation. Concurrently, decreasing $\delta^{13}C$ values point to a reduction in precipitation seasonality and enhanced C3 vegetation cover, implying a progressive reorganisation of the regional hydroclimate.

To explore the potential role of large-scale monsoon dynamics in this seasonal restructuring, we calculated the difference between unit-scale normalised (0–1) alkenone- and CCaT-based SST estimates (ΔSST_{norm}). We assume alkenone-based SST as an endmember for SAWM, and CCaT-based SST as an endmember for SASM, and thus we assume that ΔSST_{norm} reflects the overall pattern of annual monsoon wind dynamics (higher values suggest lower SAM intensity). We, however, stress that ΔSST_{norm} is not a direct or quantitatively calibrated measure of SASM versus SAWM wind strength. While alkenone-derived temperatures provide robust SST estimates, their translation into winter wind intensity remains indirect. Likewise, GDGT cyclization, and thus CCaT, likely integrates a partial thermal component in addition to influences from water-column oxygenation. Accordingly, ΔSST_{norm} should be regarded as a heuristic index capturing large-scale changes in upper-ocean and atmospheric configuration, rather than a direct measure of seasonal wind intensity.

Despite these limitations, we show that variations in ΔSST_{norm} closely parallel the divergence between unit-scale normalised leaf-wax δD and negative $\delta^{13}C$ (Supplementary Fig. 2). Within an endmember framework, this isotope relationship reflects precipitation seasonality, with lower values indicating an increased contribution of winter rainfall. Periods characterised by higher ΔSST_{norm}, implying weaker net annual monsoon circulation and coincide with isotope evidence for enhanced winter precipitation. This covariation supports the interpretation that reduced winter monsoon strength was be associated with increased winter rainfall, consistent with a shift toward stronger influence of westerly-derived moisture. Together, these observations suggest that the long-term isotopic trends primarily record a gradual rebalancing of seasonal moisture sources during the deglaciation, driven by large-scale atmospheric reorganisation, rather than changes in total or summer precipitation alone. In particular, higher ΔSST_{norm} implies lower annual net monsoon strength during the GI-1a-c, where the isotope difference suggests a stronger contribution of winter precipitation. Furthermore, a fairly negative δD during the GS-1 coupled with the most negative $\delta^{13}C$ values in our record indicates that this stadial was characterised by low

summer but increased winter precipitation, leading to expansion of C_3 plants with depleted Mediterranean-sourced δD signals.

Supplementary Text 3

To explore whether the proposed winter season bias in the isotope-based proxy record towards the end of GI-1 and GS-1 is genuine or if the increase in summer precipitation toward the end of GI-1 is more realistic, we conducted a proxy–model comparison. The results are conflicting and depend on the climate model used. TraCE-21ka[10] indicates wetter summer conditions during the early GI-1 than late GI-1 (Extended Data 4), while in contrast, iTRACE[11] simulates increased summer precipitation and more negative model-based δD values toward the GI-1a (Extended Data 5), aligning with the proxy-based δD trend. Nevertheless, it is worth noting that the increased monsoon precipitation in iTRACE is primarily related to rising greenhouse gas concentrations (Supplementary Fig. 3), rather than a northward shift of the ITCZ (Supplementary Fig. 4).

Supplementary Figures

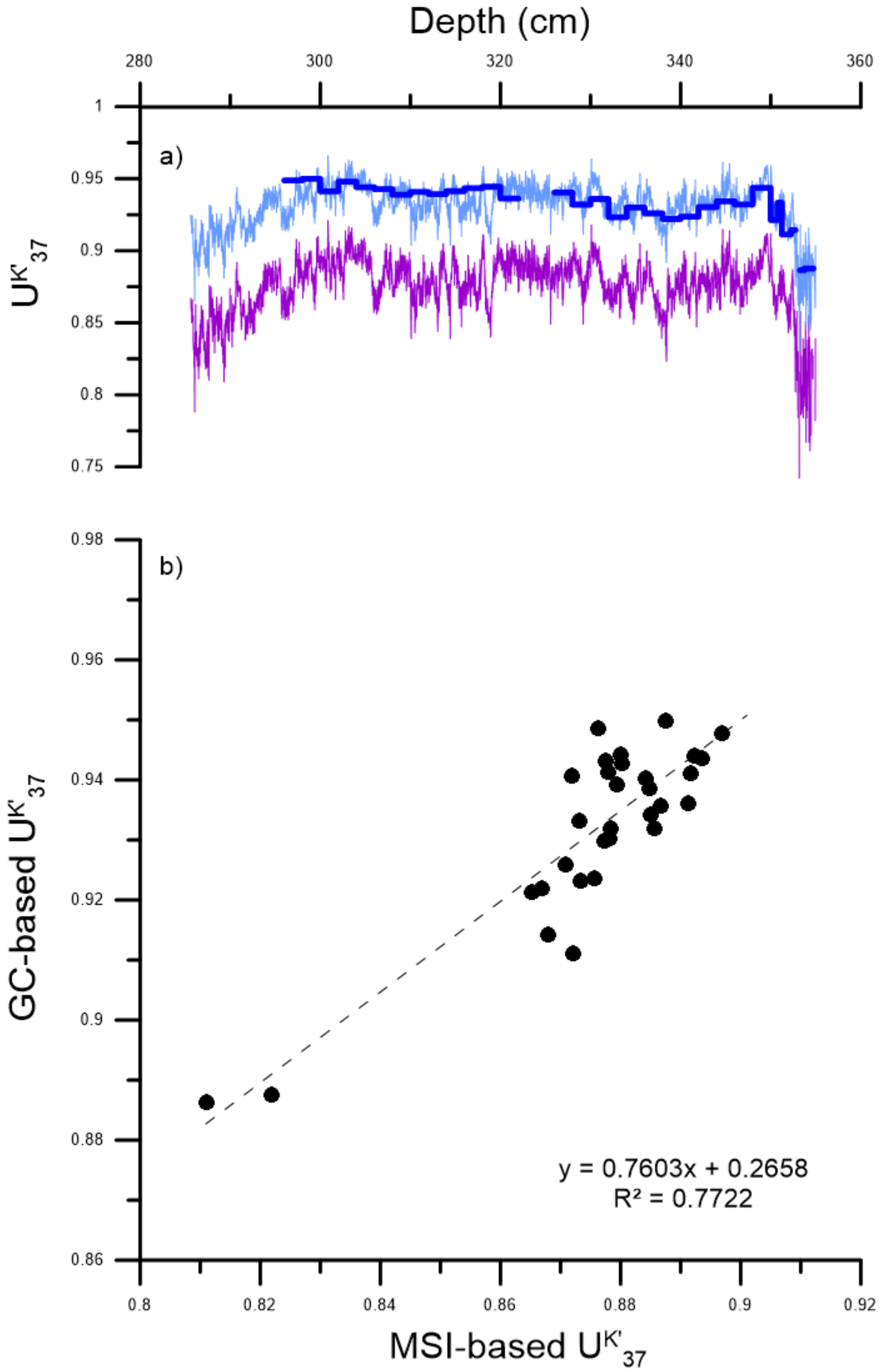

Supplementary Figure 1. $U^{K'}_{37}$ correction of MSI-based data to gas chromatography (GC)-based data. a) GC-based $U^{K'}_{37}$ (thick blue line), original MSI-based $U^{K'}_{37}$ (purple line), and corrected MSI-based $U^{K'}_{37}$ (light blue line). Regression plot of GC-based and MSI-based $U^{K'}_{37}$ for samples on the same depths. MSI data is converted to quasi-GC (corrected MSI) data following the bottom right formula, see Methods.

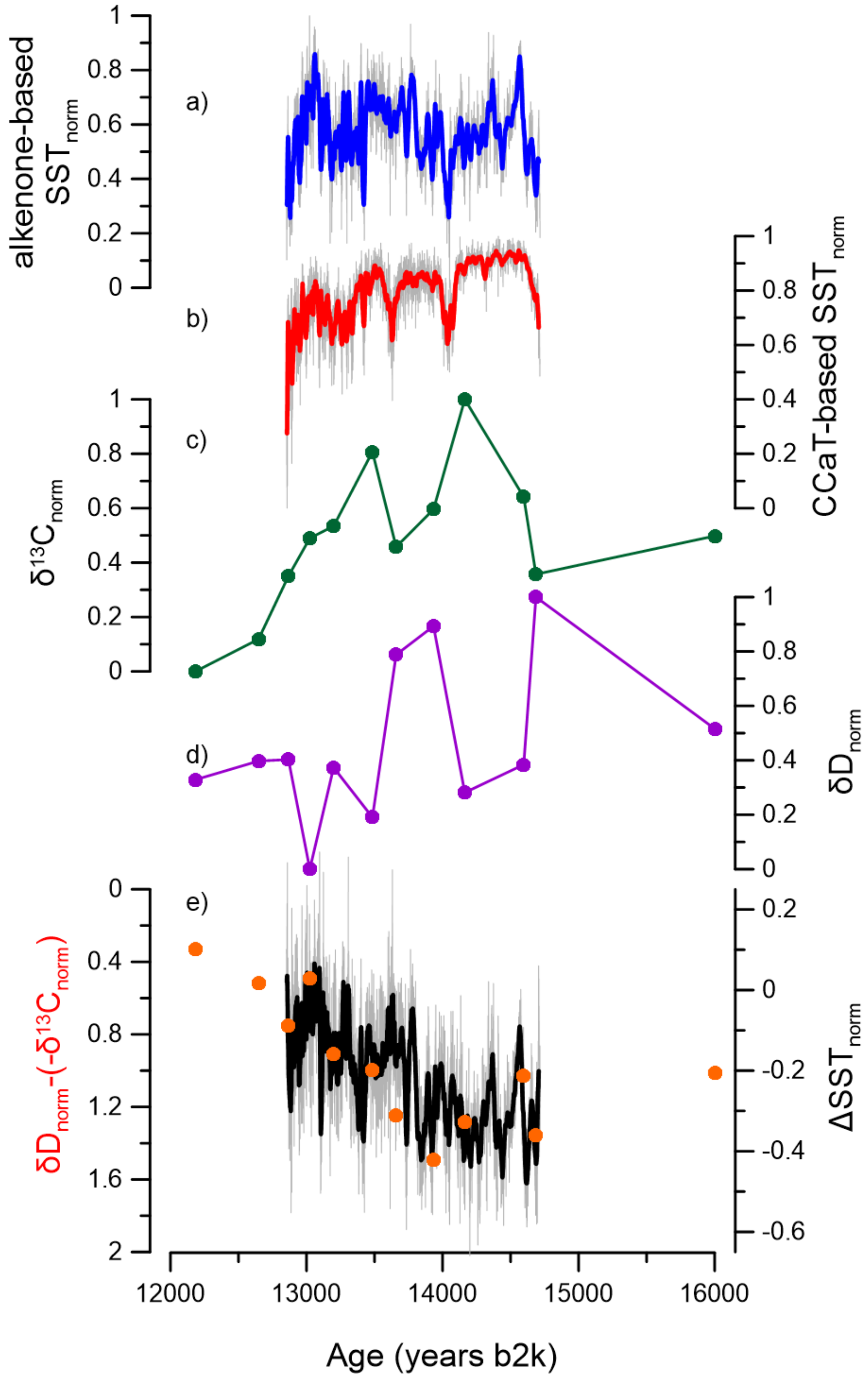

Supplementary Figure 2. Difference between unit-scale normalised alkenone- and CCaT-based SST records (ΔSST_{norm}) compared with the difference between unit-scale normalised δD and negative $\delta^{13}C$. a) Unit-scale (0-1) normalised alkenone-based SST in gray, thick blue 100-point running average. b) Unit-scale normalised CCaT-based SST in gray, thick red 100-points running average. c) Unit-scale normalised $\delta^{13}C$ and d) normalised δD. e) ΔSST_{norm} as gray line, thick black line is 100-points running average; red circles are the difference between unit-scale normalised δD and negative $\delta^{13}C$; negative $\delta^{13}C$ is applied since on millennial time scales increase in summer precipitation leads to a decrease in δD and an increase in $\delta^{13}C$.

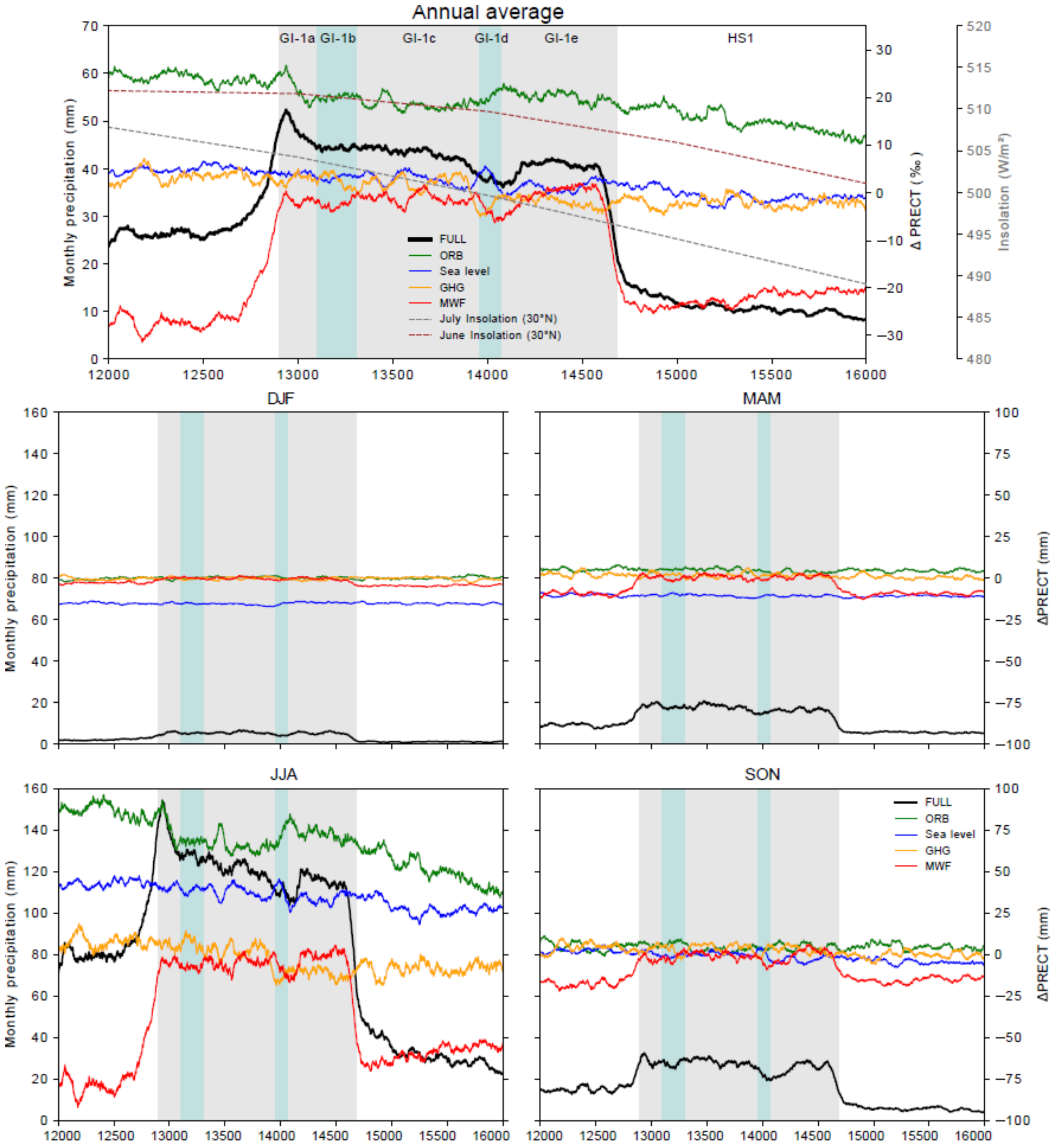

Supplementary Figure 3. Sensitivity experiments in iTRACE, showing the change caused by different forcings. Black: full forcing experiment with all forcings combined, green: orbital forcing, blue: sea level and ice sheet configuration, orange: greenhouse gases and red: meltwater forcing. Top: annual precipitation. Middle: winter (DJF) and spring (MAM) precipitation. Down: summer (JJA) and autumn (SNO) precipitation. The full forcing precipitation is on the left axes, and the ΔPRECT is the change in precipitation amount caused by the isolated forcing.

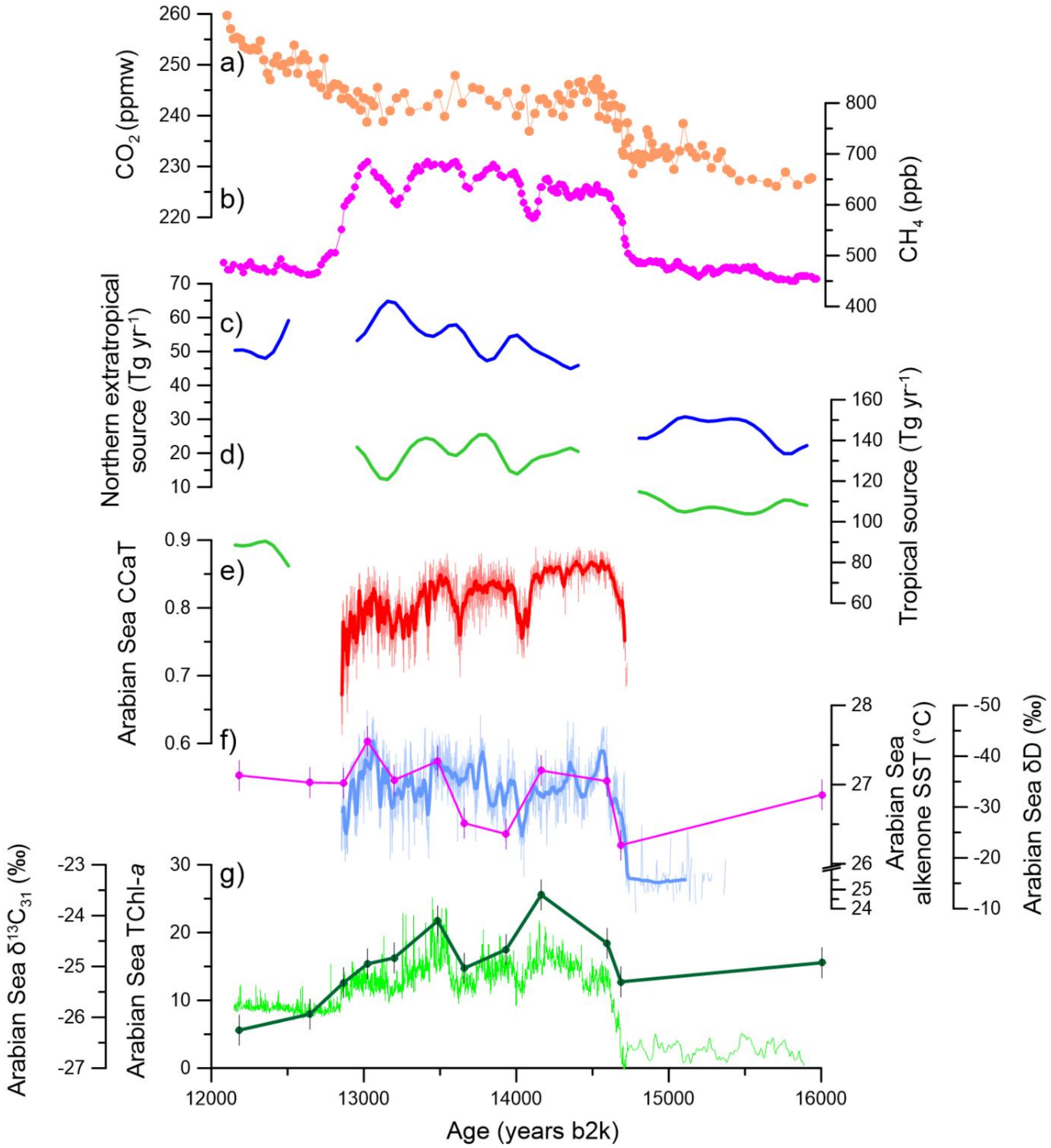

Supplementary Figure 4. Arabian Sea proxy record comparison with the deglacial greenhouse gases concentration. a) Atmospheric CO_2 and b) CH_4 concentrations from the West Antarctic Ice Sheet Divide ice core (WDC)[12], CH_4 rIPD box-model results for source strengths for the c) northern extratropical (blue) and d) total tropical (green)[13]. Note that the progressive increase in the atmospheric CH_4 during the GI-1 is driven by the Northern Hemisphere extratropical sources, highlighting that the tropical regions did not cause such a progressive increase, supporting that the ITCZ did not progressively move to the North. e) CCaT (thick red line is 50-data point running average). f) Alkenone-based SST (thick blue line is 50-data point running average) and calibration uncertainty is ±1.4°C. δD of precipitation. g) Total chlorophyll-*a* concentrations and $\delta^{13}C$ values of *n*-C_{31} alkanes.

Supplementary references

1. Napier, T. J. *et al.* Sub-annual to interannual Arabian Sea upwelling, sea surface temperature, and Indian monsoon rainfall reconstructed using congruent micrometer-scale climate proxies. *Paleoceanogr. Paleoclimatology* **37**, e2021PA004355 (2022).
2. Qin, W. *et al.* Confounding effects of oxygen and temperature on the TEX_{86} signature of marine Thaumarchaeota. *Proc. Natl. Acad. Sci. USA* **112**, 10979–10984 (2015).
3. Zhang, Z., Smittenberg, R. H. & Bradley, R. S. GDGT distribution in a stratified lake and implications for the application of TEX_{86} in paleoenvironmental reconstructions. *Sci. Rep.* **6**, 34465 (2016).
4. Elling, F. J. *et al.* Influence of temperature, pH, and salinity on membrane lipid composition and TEX_{86} of marine planktonic thaumarchaeal isolates. *Geochim. Cosmochim. Acta* **171**, 238–255 (2015).
5. Evans, T. W. *et al.* Lipid biosynthesis of *Nitrosopumilus maritimus* dissected by lipid-specific radioisotope probing (lipid-RIP) under contrasting ammonium supply. *Geochim. Cosmochim. Acta* **242**, 51–63 (2018).
6. Hurley, S. J. *et al.* Influence of ammonia oxidation rate on thaumarchaeal lipid composition and the TEX_{86} temperature proxy. *Proc. Natl. Acad. Sci. USA* **113**, 7762–7767 (2016).
7. Zander, P. D., Wienhues, G. & Grosjean, M. Scanning hyperspectral imaging for in situ biogeochemical analysis of lake sediment cores: review of recent developments. *J. Imaging* **8**, 58 (2022).
8. Polik, C. A., Elling, F. J. & Pearson, A. Impacts of paleoecology on the TEX_{86} sea surface temperature proxy in the Pliocene–Pleistocene Mediterranean Sea. *Paleoceanogr. Paleoclimatol.* **33**, 1472–1489 (2018).
9. Tierney, J. E., Pausata, F. S. R. & deMenocal, P. Deglacial Indian monsoon failure and North Atlantic stadials linked by Indian Ocean surface cooling. *Nat. Geosci.* **9**, 46–50 (2016).

10. He, F. Simulating transient climate evolution of the last deglaciation with CCSM3. PhD thesis, 642 University of Wisconsin–Madison, Department of Atmospheric and Oceanic Sciences (2011).

11. He, C. Deciphering the deglacial evolution of water isotope and climate in the Northern Hemisphere. PhD thesis, The Ohio State University, School of Earth Sciences (2021).

12. Marcott, S. A. et al. Centennial-scale changes in the global carbon cycle during the last deglaciation. Nature 514, 616–619 (2014).

13. Riddell-Young, B. et al. Atmospheric methane variability through the Last Glacial Maximum and deglaciation mainly controlled by tropical sources. Nat. Geosci. 16, 1174–1180 (2023).
