# Supplementary material for: Contrasting drivers of South Asian summer and winter monsoon evolution during the last deglaciation
Source: Nat Geosci. 2026 Jun 22;19(8):959–65. doi: 10.1038/s41561-026-02023-z (PMC13447086; doi:10.1038/s41561-026-02023-z)
Supplement: Supplementary file 2 [file 41561_2026_2023_MOESM2_ESM.pdf]

nature portfolio

Peer Review File

Contrasting drivers of South Asian summer and winter monsoon evolution during the last deglaciation

Corresponding Author: Professor Igor Obreht

Version 0:

Reviewer comments:

Reviewer #1

(Remarks to the Author)
Over recent decades, a great deal of attention has been paid to the evolution of the Asian summer monsoon. The winter monsoon, being more difficult to reconstruct and perhaps less critical to regional ecology and socioeconomics, is less well understood. This study aims to resolve both. High resolution imaging of a laminated section of an Arabian Sea core spanning a short period (GI-1) of the last deglaciation is coupled with carbon isotopic analyses and paleo-SST reconstructions. The results are modestly interesting/novel, particularly the discussion of the SAWM, but the time interval of interest is quite short and the text jumps from one subtopic to the next without the necessary amount of explanation. As a result, I found it difficult to follow the thread of the storyline.

The explanation of the Indian summer monsoon is not well explained and a bit oversimplified. At one point, it is discussed as a sea breeze analogue, while later it is presented as representing a manifestation of the ITCZ. The reality is in between.

The chronology is not explained in any meaningful way, nor is the tuning of the age model to the Greenland ice record (which itself is tuned to the Chinese stalagmite record, I believe). This omission is quite unusual, and in fact I can't remember seeing a manuscript that doesn't present the details of its chronology. This raises a red flag for me.

Controls on precipitation d18O (dD) are oversimplified. Amount effects on precipitation d18O are present in some areas of South Asia but broadly absent in many others. Monsoon precip is generally isotopically lighter than non-monsoon precip. Recycling of monsoon rainfall discharge into the Indian Ocean amplifies reductions in monsoon rainfall d18O in the latter part of the monsoon season.

With respect to the SAWM, interpretations of the southern China maar record cited here have been challenged. But even so, its location puts it into the region impacted by winter outflows associated with the Siberian High. It is unclear how/why the marine record here, being located so much further west, would align. Also, Northern Hemisphere DJF insolation is falling during the last deglaciation. Would not this have strengthened the SAWM?

20 – delete "for"
21 – "sufficiently high" to meet what goal?
27 – why is it important to quantify this SST range here?
29 – this finding doesn't seem particularly novel; the speleothem records have demonstrated this for decades.
30 – what is meant by "long term"?
32 – this is more novel and interesting
34 – this abstract needs some attention; it's not particularly clear
40 – this "sea breeze" model for the monsoon is oversimplified. The ITCZ is mentioned later in a way that also is oversimplified.
49 – the introduction is much better written than the abstract
50 – awkwardly worded sentence
53 – what about the end of the Younger Dryas at 11,600 yr ago?
56 – again, the speleothem records preserve precisely-dated, sub-decadal information on periods of profound and rapid environmental change
65 – I don't understand what is meant by "govern evaporation rates". Evaporation would be reduced in both surface water and air temperatures are low. It would seem that higher SSTs and air temps in summer, particularly prior to the start of the monsoon, would yield much higher evap rates. And how do winter temps determine "annual" SST?
89 – as in the abstract, SST are quantified here. It is unclear what the reader is to take from this as they are within error of each other and temperatures in HS1, the YD, or the modern are not presented here.

121 – it's been a while, but I believe some of the interpretations of the Huguang Maar SAWM reconstruction have been called into question by Zhou (2009) Quat. Res. and Shen (2013) GRL
130 – a solar influence has been demonstrated in other South Asian archives, but why only a multi-centennial influence here? Where are the shorter frequencies?
163 – it is true that winter precip can influence mean annual d18O (or dD) values, but as these represent small contributions to total annual rainfall, large changes in winter precip are required to make a noticeable dent in the overall isotope ratio. Reference 40 suggests winter rains can account for up to 33% in northern India but many parts of India receive a small percentage in winter.
164 – These d13C values seem pretty consistent with C3 plants, and yet they are being used to support the conjecture that C4 plants were present in the Indus River catchment. The shifts are also very small (~1‰). a
178 – this seems a bit confusing as monsoon rains should have a lower d18O than non-monsoon winter rains.
288 – The chronology is explained using two sentences in the Methods. The first is "The first-order age model for the entire 20.2 m SO130-289KL core is based on 20 accelerator mass spectrometry 14C dates and cross-correlation to radiocarbon dated constrained by 7 14C dates." I've never read a manuscript where no further mention of radiometric dates is made beyond stating they were obtained. Are they robust? Which lab did the work? What materials were dated? Do they fall in stratigraphic order? Where any exlcluded from consideration? If so, why? It's also unclear what is meant by what appears to be the incomplete phrase "and cross-correlation to radiocarbon dated constrained by 7 14C dates". Is there a word missing after dated? How do the 7 14C dates differ from 20 AMS dates?

Reviewer #2

(Remarks to the Author)
This is a promising high-quality paper with impressive methodological advancements and high-quality data.

Reviewer #3

(Remarks to the Author)
The manuscript submitted by Obreht et al. for publication in Nature Geoscience presents a unique and exceptionally valuable set of ultra–high-resolution data derived from laminated sediments of the northern Arabian Sea. These records are used to investigate South Asian monsoon dynamics at an unprecedented temporal resolution across the GI-1 interval. The study places particular emphasis on the South Asian winter monsoon, which has received far less attention than the summer monsoon due to the dominance of the latter in most sedimentary and speleothem archives.
The manuscript is clear and well written. The originality of the data set, together with its innovative use to decipher decadal- to millennial-scale variability in past monsoon systems, strongly supports publication in a journal such as Nature Geoscience. I have only a limited number of relatively minor comments and questions, which should be straightforward for the authors to address.

Line 41 – The statement is not fully correct. Most aeolian dust deposited over the Arabian Sea originates from the Arabian Peninsula and adjacent northwestern regions (Iran–Pakistan) and is transported primarily by northerly to northwesterly winds, often above the summer monsoon layer. These include Shamal-type flows and other dry continental air masses. In contrast, low-level southwest monsoon winds are humid and oceanic in origin and therefore play a secondary, mainly redistributive role rather than acting as the primary dust source.

Line 45 – The authors should already mention here that marine sedimentary records of paleo-monsoon from the Arabian Sea are largely dominated by the South Asian Summer Monsoon (SASM) signal (productivity-related records, upwelling).

Figure 1 – The authors should specify the altitude level at which the back trajectories were computed (e.g., 850 hPa, which is traditionally used for monsoon wind analyses).

In addition, the rationale for selecting an inland reference point for back-trajectory calculations is unclear. Although backtracking from this location highlights the summer circulation pattern, it is less effective in illustrating the northeast wind regime over the Arabian Sea. A reference point located near the northeastern boundary of the Arabian Sea might have provided a more representative depiction of winter circulation. If the inland position was chosen to characterize moisture transport within the Indus catchment and its relevance to the Bittoo and Timta cave records, this motivation should be explicitly stated in the manuscript. Incidentally, enlarging the map would also allow the authors to indicate the locations of the cave and maar records used in Figure 3.

Line 54 – The authors correctly describe the studied interval as a natural experiment for investigating the evolution of the South Asian Monsoon (SAM) system during abrupt climate changes. However, they do not sufficiently emphasize here that these rapid climate events occurred within a specific climatic context, namely increasing Northern Hemisphere summer insolation, the onset of the last major deglaciation, and high-amplitude millennial-scale climate oscillations characteristic of glacial conditions. While the limited contextualization at the beginning of the paper does not constitute a strong weakness — given that the authors aim primarily to provide high-resolution insights into monsoon dynamics and not an analogue for modern warming — the broader climatic framework should nevertheless be more clearly articulated from the start.

Line 73 – The authors report using the Uk37 alkenone unsaturation index and the GDGT-based Crenarchaeol–Caldarchaeol Tetraether (CCaT) index as two independent SST proxies. However, they later acknowledge that CCaT is influenced by GDGT cyclization, which responds to oxygen availability and OMZ intensity associated with upwelling in the western Arabian Sea. I suggest that (1) the authors explicitly state from the outset that CCaT is affected by cyclization in this

region (with a brief one-sentence explanation of cyclization), and (2) directly reference previous work (e.g., Napier et al.) demonstrating that CCaT at this site is better interpreted as a proxy for oxygenation and, by extension, productivity rather than SST.

Accordingly, the raw CCaT index should be better plotted in Figure 2, as is done in Figure 3, instead of presenting CCaT-derived temperatures.

Line 105 – Overall, the deglacial CCaT record aligns well with Greenland ice-core $\delta^{18}O$ (Fig. 2), except toward the end of the sequence. Specifically, CCaT declines from the GI-1b/GI-1a transition upward —consistent with the reflectance record and alkenone-based SST— whereas Greenland $\delta^{18}O$ continues to rise before decreasing later during GI-1a. Moreover, the transition to GS-1 corresponds to a sharp decrease in CCaT, with an amplitude comparable to the CCaT increase observed at the HS1–GI-1 transition. In contrast, Greenland $\delta^{18}O$ exhibits a more gradual and lower-amplitude change at the onset of GS-1, unlike the abrupt and large-amplitude shift at the HS1–GI-1 transition. An age-model issue to explain a possible mismatch between the two records seems unlikely, as the SO130-289KL chronology was fine-tuned to Greenland ice-core records. Could the authors provide an explanation for the apparent anti-correlated behavior between CCaT and Greenland $\delta^{18}O$ during early GI-1a, as well as for the discrepancy in signal amplitude?

Line 119 – Typographical error: “late stage of GS-1” should read “late stage of GI-1”.

Line 125 – Wavelet spectral analysis indicates a significant in-phase relationship between SST and CCaT on multidecadal timescales during GI-1a and GI-1b. This covariance is also evident from visual inspection and resembles the behavior observed during GI-1d, where decreases (increases) in SST coincide with decreases (increases) in CCaT. Does this imply that SASM and SAWM wind intensities co-varied during these intervals?

Line 133 – The authors conduct cross-wavelet analyses between Arabian Sea alkenone-based SST, CCaT, and Greenland ice-core 10 Be flux to test whether the 200–250-year periodicity could be linked to solar variability. They report a significant in-phase relationship within this band. Is this zero phase relationship the principal argument for a solar forcing mechanism? Given that the SO130-289KL chronology is tuned to Greenland ice-core records, lead–lag relationships between NGRIP and marine proxies cannot be meaningfully interpreted.

Line 160 – The authors state that δD values between –155‰ and –136‰ reflect precipitation dynamics within the Indus River catchment, the primary source of leaf-wax n-alkanes. However, Figure 3 shows δD values ranging only from approximately –10‰ to –50‰. This discrepancy should be clarified.

Moreover, δD values do not unambiguously distinguish wetter interstadial from stadial conditions, as GS-1 values overlap substantially with those from the GI-1 interval.

Line 169 – Typographical error: “increase in southeast SASM winds” should read “increase in southwest SASM winds”.

Line 174 – The authors describe a regime shift beginning in GI-1b, characterized by concurrent decreases in $\delta^{13}C$ and δD. However, in my view, the records instead appear to display a broader-scale trend of progressively decreasing $\delta^{13}C$ and δD from HS1 to GS-1, upon which two centennial- to millennial-scale oscillations during GI-1 are superimposed. If confirmed by higher-resolution data, this long-term evolution would suggest a progressive response to gradual forcing mechanisms, such as increasing insolation and/or the ongoing deglaciation. Notably, a trend toward lighter $\delta^{13}C$ values in n-C_{31} alkanes—implying an increased contribution of C_4 plants—would not be straightforwardly compatible with a global increase in insolation-driven seasonality. As pointed out by the authors (ie line 193), an important component of the δD decrease may instead reflect the progressively increasing influence of isotopically depleted Mediterranean-sourced winter moisture during the last deglaciation. The authors are encouraged to discuss the possibility and underlying mechanisms of a long-term trend in δD and $\delta^{13}C$ over the full duration of the records.

Line 197 – I am not convinced by the strategy adopted by the authors. While alkenone-derived temperatures represent robust, well-calibrated SST estimates, their empirical translation into winter wind intensity is far less secure. Furthermore, the authors hypothesize —based on a single published study— that CCaT variability is primarily controlled by GDGT cyclization and therefore does not robustly record temperature, but instead reflects changes in OMZ oxygenation and, by extension, productivity. Even if this interpretation is correct, CCaT would remain a highly indirect and non-calibrated proxy that is only loosely related to summer wind intensity. Consequently, any attempt to quantify the relative strength of SASM versus SAWM by differencing these proxies appears questionable.

Version 1:

Reviewer comments:

Reviewer #1

(Remarks to the Author)
I reviewed an earlier version of this manuscript and had several concerns. The authors have done a fine job of addressing the majority of these and I feel that this is a sufficiently well-constructed and innovative study to merit publication in Nature Geoscience. The only substantive issue remaining is the decision to use end-member rather than mean d13C values for C3

and C4 vegetation (see line 175). This decision seems to have been made to fit the data to the interpretation rather than the other way around and as such, it undermines an otherwise solid paper.

I include a few very minor word-smithing suggestions below.
38 – please reword “the poleward of equator”
41 – perhaps “drier” since part of the point is that some precip is associated with the SAWM
44 – “increased”?
48 – “sparsity”?
50-57 – “abrupt” is used a lot here. Reword?
67 – no comma after cold
78 – proximal instead of close-by?
116 – trends?
129-136 – “multidecadal” is used a lot here. Reword?
141 – Therefore?
147-with the 10Be?
228 – precipitation, but similar to?

Reviewer #3

(Remarks to the Author)
In my view, the authors have responded satisfactorily to my questions and comments and have improved the quality of the manuscript compared to its initial version. I therefore support the publication of this work in Nature Geoscience.

Version 2:

Reviewer comments:

Reviewer #1

(Remarks to the Author)
The minor edits I requested have been fully addressed, and the manuscript is now suitable for publication.

Open Access This Peer Review File is licensed under a Creative Commons Attribution 4.0 International License, which permits use, sharing, adaptation, distribution and reproduction in any medium or format, as long as you give appropriate credit to the original author(s) and the source, provide a link to the Creative Commons license, and indicate if changes were made.
In cases where reviewers are anonymous, credit should be given to 'Anonymous Referee' and the source.
The images or other third party material in this Peer Review File are included in the article’s Creative Commons license, unless indicated otherwise in a credit line to the material. If material is not included in the article’s Creative Commons license and your intended use is not permitted by statutory regulation or exceeds the permitted use, you will need to obtain permission directly from the copyright holder.

To view a copy of this license, visit https://creativecommons.org/licenses/by/4.0/

Dear Professor Obreht,

Your manuscript entitled "South Asian summer and winter monsoon evolution during the last deglaciation" has now been seen by 3 reviewers, whose comments are included below. I'm sorry for the delay in sending you this update, which was due to difficulties securing reviewer reports. Although they find your work of some potential interest, they have raised concerns which in our view are sufficiently important to preclude publication of the work in Nature Geoscience, at least in its present form.

We thank the Editor for the opportunity to revise and resubmit our manuscript. Following the editorial and reviewers' suggestions, we have substantially improved the study, where the revised manuscript now more clearly and robustly conveys our main results and conclusions regarding the decadal-to-millennial scale dynamics of the summer and winter South Asian monsoon during the last deglaciation.

We are particularly concerned that the referees are not fully convinced that your conclusions about the decadal-to-millennial scale dynamics of the Summer and Winter Asian Monsoon the last deglaciations have been robustly and clearly justified. The nature of the criticisms raised makes it hard for us to envisage that a straightforward revision of the present manuscript would be likely to allow you to strengthen your case sufficiently.

If, after future work, you can provide more compelling support that

1) that assumptions in modelling and biomarker approach are fully supported and that all relevant uncertainties are accounted for in the interpretations,

In our detailed response to the reviewers, we have provided a thorough justification of the assumptions underlying both the biomarker and modelling approaches. This includes examples of different back-trajectory scenarios, a clear discussion of the uncertainties associated with our estimates, and an explicit explanation of the benefits and limitations of our approach. These considerations ensure that all relevant uncertainties are transparently addressed in our interpretations.

2) that suitable calibrations and age constraints are used and presented,

Regarding the age control, we clarified that the age model was robustly established in the previous work (Deplazes et al., 2013), which was previously published in Nature Geoscience. We supplement reviewers' rebuttal with all necessary information and updated our method section (lines 297-303).

3) that differences in the intensity of the winter and summer monsoons can be clearly distinguished and that the drivers of these trends through the study interval have been compellingly identified,

In response to the reviewers' comments, we have strengthened the interpretation of our multiproxy dataset, showing more clearly that it robustly distinguishes summer and winter monsoon intensity. We incorporated Greenland summer temperature model-proxy estimates at the ice core sites, which show stronger correspondence with our South Asian summer monsoon proxies, supporting the role of Northern Hemisphere high-latitude climate forcing in driving summer monsoon variability (newly generated Extended Data 1 and lines 109-111). We also clarified the increased winter precipitation in our records. In particular, we addressed Reviewer 1's question about whether winter or summer precipitation is more depleted (lines 195-202), highlighted that Greenland Stadial 1 coincided with enhanced winter precipitation as suggested by Reviewer 3 (lines 191-195), and noted that winter precipitation can be influenced by temperature effects, unlike summer monsoon rainfall (230-233). Together, these revisions support the interpretation that winter monsoon rainfall reflects a long-term orbital-scale warming trend, modulated by millennial-scale Northern Hemisphere variability.

then we would be pleased to consider a revised manuscript (unless, of course, something similar has by then been accepted at Nature Geoscience or appeared elsewhere).

Reviewers' comments:

Reviewer #1 (Remarks to the Author):

Over recent decades, a great deal of attention has been paid to the evolution of the Asian summer monsoon. The winter monsoon, being more difficult to reconstruct and perhaps less critical to regional ecology and socioeconomics, is less well understood. This study aims to resolve both. High resolution imaging of a laminated section of an Arabian Sea core spanning a short period (GI-1) of the last deglaciation is coupled with carbon isotopic analyses and paleo-SST reconstructions. The results are modestly interesting/novel, particularly the discussion of the SAWM, but the time interval of interest is quite short and the text jumps from one subtopic to the next without the necessary amount of explanation. As a result, I found it difficult to follow the thread of the storyline.

We thank the reviewer for their careful reading of the manuscript and for their constructive comments. We appreciate their assessment of the novelty of the results, particularly regarding the South Asian winter monsoon. While we understand that the studied interval is relatively short, the production of high-resolution SST records requires substantial analytical effort and time. We therefore consider the duration of the record to be justified by the exceptional temporal resolution achieved and the detailed insights it provides into climate variability during this interval; as an example, for 1 cm of sediment, which is typically considered as a high sampling resolution, we provide 50 datapoints with average temporal resolution of 0.6 years. Moreover, we acknowledge that the original version of the manuscript lacked sufficient explanation in places, which made the overall storyline difficult to follow. We believe that the revised version addresses the reviewer's concerns and is now easier to follow, see also our detailed response below.

The explanation of the Indian summer monsoon is not well explained and a bit oversimplified. At one point, it is discussed as a sea breeze analogue, while later it is presented as representing a manifestation of the ITCZ. The reality is in between.

This is a valid point and in the revised version, we better explained our present-day understanding of the summer monsoon dynamics (e.g. lines 37-39).

The chronology is not explained in any meaningful way, nor is the tuning of the age model to the Greenland ice record (which itself is tuned to the Chinese stalagmite record, I believe). This omission is quite unusual, and in fact I can't remember seeing a manuscript that doesn't present the details of its chronology. This raises a red flag for me.

The reason to omit a detailed presentation or discussion of the chronology in the original version was that the age model for this core had been previously established in Deplazes et al. (2013) and was independently tuned to the Greenland ice core record. This tuning is further supported by its coherence with the reflectance record of Cariaco Basin, as presented in the same study (Deplazes et al., 2013), which serves as a key marine reference for radiocarbon calibration beyond 14 ka (Hughen et al., 2006; Hughen et al., 1997; Hughen & Heaton, 2020). We note that the tuning to the ice core records was carried out for the establishment of absolute chronology, and for the scope of this study this falls within deglaciation, where Greenland ice chronology uncertainty is limited to decades (Adolphi et al., 2018). We also highlight that the Greenland ice cores are independently layer-counted and no tuning to speleothems is involved, for comparison between speleothems and ice cores please see (Adolphi et al., 2018; Sinnl et al., 2023). We highlight that one of the primary reasons for selecting this site for high-resolution analyses was the robustness and reliability of its established chronology. Nonetheless, to improve clarity and transparency, the revised manuscript now provides an expanded description of the age model (See Methods).

Controls on precipitation d18O (dD) are oversimplified. Amount effects on precipitation d18O are present in some areas of South Asia but broadly absent in many others. Monsoon precip is generally isotopically lighter than non-monsoon precip. Recycling of monsoon rainfall discharge into the Indian Ocean amplifies reductions in monsoon rainfall d18O in the latter part of the monsoon season.

We thank the reviewer for highlighting the complexities in interpreting precipitation δ¹⁸O and δD in South Asia. We fully acknowledge that monsoon precipitation is generally isotopically lighter than pre- and post-monsoon precipitation in much of the Asian monsoon region, and that simple amount effects cannot fully explain observed isotope variability; this is also the central premise of our interpretation.

Our interpretation does not rely on non-monsoon precipitation being fundamentally more depleted than summer monsoon rainfall across the region. However, winter precipitation can be isotopically lighter under certain conditions, such as from moisture sourced from distant regions (e.g., Mediterranean), or at high elevations, where lower condensation temperatures and snow formation produce more depleted δD values. In fact, in the area of the upper Indus catchment in Tibetan Plateau, winter precipitation is significantly more depleted than summer (Zhu et al., 2023), albeit this is valid for only one part of the Indus catchment. Such isotopically depleted (winter) precipitation can contribute disproportionately to the integrated isotope signal in South Asia. During periods of reduced seasonality, such as late Greenland Interstadial 1 and Stadial 1, decreases in summer monsoon precipitation may not be fully captured in the δD record if the relative contribution of winter precipitation increases. In this context, enhanced winter precipitation can partially compensate for reductions in summer rainfall, modifying the integrated δD signal recorded in the Arabian Sea and δ¹⁸O signal in speleothems. Therefore, we argue that the observed decrease in δD during Greenland Interstadial 1a-b and Greenland Stadial 1 reflects changes in seasonal moisture balance and circulation, rather than implying an increase in summer monsoon precipitation. This emphasizes that the Arabian Sea isotope record integrates contributions from multiple seasons and moisture sources over the Indus River Basin, highlighting the importance of considering both summer monsoon and winter precipitation in hydroclimate reconstructions. We also agree that post-monsoon precipitation is influenced by moisture recycling. However, local moisture recycling is unlikely to dominate winter precipitation in the Indus Valley, given the large volumes required to feed snowpacks and glaciers and the frequency of heavy winter precipitation events (Hunt et al., 2025). However, we rephrased statements in our manuscript to avoid confusion that we claim that winter precipitation is generally more depleted than summer, see lines 195-198.

With respect to the SAWM, interpretations of the southern China maar record cited here have been challenged. But even so, its location puts it into the region impacted by winter outflows associated with the Siberian High. It is unclear how/why the marine record here, being located so much further west, would align. Also, Northern Hemisphere DJF insolation is falling during the last deglaciation. Would not this have strengthened the SAWM?

We thank the reviewer for highlighting this point. We do not intend to overinterpret our data and have deliberately toned down our implications in the manuscript (lines 248-251).

However, we also highlight that there are substantial disagreements between isotopic and non-isotopic proxies in Asian monsoon hydroclimate records, with rainfall isotopes repeatedly shown to reflect processes other than the summer monsoon amount effect (Jing et al., 2025). We also agree that winter precipitation is not a main driver of isotope signal in the southern China. Nonetheless, winter precipitation in this region is largely accosted with wind trajectories originating from Mediterranean, see Fig. R1 for the example of the Mawmluh cave. Nonetheless, under current climatic conditions, the isotopically depleted winter precipitation from western disturbances influences δD signal in South Asia, but not southern China, as correctly pointed out by the referee. However, periods with reduced SASM and increased winter precipitation when compared to present day, as e.g. GS-1, could be more sensitive to winter rainfall. Moreover, the observed resemblance between our δD record and East Asian winter proxies reflects the potential large-scale influence of westerly jet streams, high-pressure systems, and orbital forcing on winter precipitation, rather than a direct effect of regional western disturbances. We also point out that winter outflows associated with the Siberian High would also contribute with depleted isotopic signal. Nonetheless, this goes beyond the scope of our study, and we included this statement in the manuscript: “While there are broad similarities between our δD record and other Asian monsoon proxies, the seasonal bias mechanism proposed here cannot be directly

applied to the entire Asian Monsoon system, and further research is needed to assess the role of large-scale atmospheric forcing on winter precipitation across these regions.”

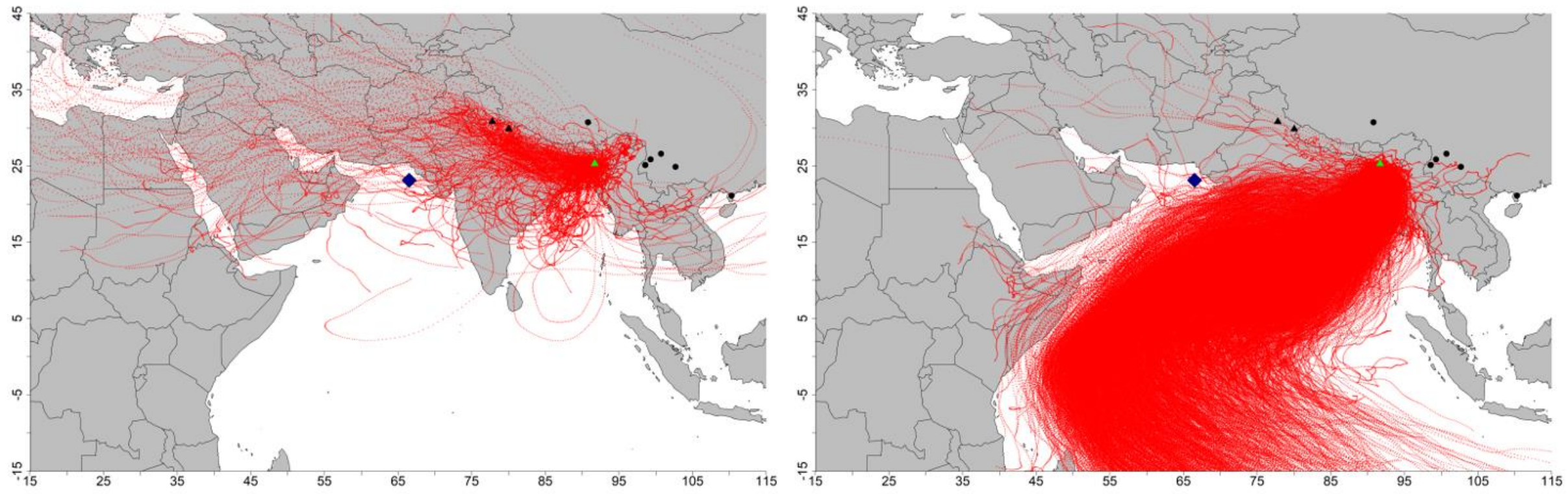

Figure R1. Seasonal back trajectories for the region which includes the location of the Mawmluh Cave. Left: Plot of the back trajectories for 240 h during winter. Right: Plot of the back trajectories for 240 h during summer.

Moreover, we also point out the temperature effect would have a much stronger effect on winter precipitation, which could potentially explain larger scale pattern. While large-scale interpretation goes above the scope of our study, we did include part in lines 230-235: Moreover, we emphasize that the effect of temperature on monsoon rainfall is low; however, temperature plays a significant role in controlling the isotopic composition of precipitation associated with westerly jets. Thus, an increase in the proportion of winter precipitation additionally lowers the mean annual $\delta^{18}O$ values through the temperature effect[44].

In regard to the decreasing Northern Hemisphere DJF insolation, we argue that winter monsoon strength is primarily controlled by the land–sea thermal contrast. During the last deglaciation, long-term warming reduced this thermal gradient, leading to a weakening of the South Asian Winter Monsoon, despite the modest decline in DJF insolation.

20 – delete “for”

Done

21 – “sufficiently high” to meet what goal?

Rephrased, please see lines 20-21.

27 – why is it important to quantify this SST range here?

We agree that quantitative SST values in the abstract do not, by themselves, provide a full climatological importance of the data. We thus excluded it from the abstract. However, a key novelty of our record is precisely that it allows quantification of SST variability at an unprecedented temporal resolution, which is not possible for other paleoclimate archives because either temperature estimates are qualitative or semi-quantitative (e.g., speleothems, ice cores, or tree rings), or the temporal resolution of the records is not sufficient (e.g. marine and lake records). We therefore retain the quantified SST range in the main text to highlight this unique strength of the dataset and to emphasize the added value of our approach.

29 – this finding doesn’t seem particularly novel; the speleothem records have demonstrated this for decades.

We largely agree; however, we also show that at the centennial resolution, speleothem $\delta^{18}O$ signals are partly biased toward the winter contribution. The novelty of our study lies in producing a record that

more clearly disentangles the relative contributions of winter and summer monsoon precipitation and their evolution.

30 – what is meant by “long term”?

Rephrased to long term millennial, please see line 28.

32 – this is more novel and interesting

Thank you for comment.

34 – this abstract needs some attention; it’s not particularly clear

We thank the reviewer for pointing out that the original abstract did not coherently reflect the main messages of the manuscript. The abstract has been revised and we believe it now provides a clearer and more coherent summary of our key findings.

40 – this “sea breeze” model for the monsoon is oversimplified. The ITCZ is mentioned later in a way that also is oversimplified.

We now explain these mechanisms in a more precise, yet concise way and with a reference to the relevant review paper (lines 37-40).

49 – the introduction is much better written than the abstract

Thank you.

50 – awkwardly worded sentence

We included new sentence: Examining abrupt climate transitions in the past provides valuable insights into the response of the SAM to rapid climate change.

53 – what about the end of the Younger Dryas at 11,600 yr ago?

We agree that the termination of the Younger Dryas constitutes an abrupt climate transition, albeit this is transition into the interglacial and not DO event. However, this is not covered by our record and therefore, we change the phrasing in the revised version to: “are two of the most recent abrupt climate transitions”.

56 – again, the speleothem records preserve precisely-dated, sub-decadal information on periods of profound and rapid environmental change

We agree. We have rephrased the sentence to clarify this point: “Nevertheless, two primary challenges hinder our ability to capture paleoclimate signals at near-decadal resolution: the limited distribution of archives, such as speleothems or finely laminated sediments, that preserve undisturbed signals, and the analytical constraints inherent in conventional techniques due to sample size requirements, which are particularly pertinent for sediments.”

65 – I don’t understand what is meant by “govern evaporation rates”. Evaporation would be reduced in both surface water and air temperatures are low. It would seem that higher SSTs and air temps in summer, particularly prior to the start of the monsoon, would yield much higher evap rates. And how do winter temps determine “annual” SST?

The SAWM winds cause anomalously high latent heat loss and evaporative cooling, and thus influence the annual SST integrated over the year, as the effect of SASM in this part of the Arabian Sea is minor. To clarify, we have revised the text as follows: “During the boreal winter, strong, cold, and dry northeasterly monsoon winds increase latent heat loss and evaporative cooling at the sea surface, determining the seasonally integrated sea surface temperature (SST) of the northeast Arabian Sea. As a result, stronger SAWM winds lead to lower annual mean SSTs[8–11].”

89 – as in the abstract, SST are quantified here. It is unclear what the reader is to take from this as they are within error of each other and temperatures in HS1, the YD, or the modern are not presented here.

We thank the reviewer for the comment. We changed the phrasing to : “Alkenone-based SST indicates a prominent, abrupt increase of up to ~2°C in just a few decades during the HS1 and GI-1 transition and reached values of 27.5±1.4°C. The SSTs during the GI-1 interstadial remained high between 26±1.4°C and 27.5±1.4°C.”

It is important to clarify, however, that the reported SST uncertainty reflects absolute calibration uncertainty, not the amplitude of relative variability. In other words, while the entire time series may lie within ±1.5 °C of the “true” SST estimates, this does not imply that each individual data point can independently vary anywhere within that range. The trends themselves are robust. Unlike isotopic proxies from foraminifera or speleothems, each SST datapoint is coherently connected to preceding and following points within the uncertainty scale. This coherence has been demonstrated at subannual resolution in sediment trap data (Müller & Fischer, 2001; Rosell-Melé & Prahl, 2013), as well as in empirical (Alfken et al., 2020; Napier et al., 2022) and cultural proxy datasets (Prahl et al., 2003). Therefore, although the absolute SST estimates are less precise (within an envelope of ~3 °C), the relative variability and trends in the record are reliable. This is why we did not include a direct reference to present-day SST in the previous version, as our focus was on the relative variability. We hope that this clarifies that the variability within the uncertainty envelope is robust, even if the absolute values carry some uncertainty.

121 – it’s been a while, but I believe some of the interpretations of the Huguang Maar SAWM reconstruction have been called into question by Zhou (2009) Quat. Res. and Shen (2013) GRL

This is a fair remark, and we have addressed it in the manuscript. In the revised text, we explicitly note that the use of Huguang Maar sediments as a winter monsoon proxy remains debated and highlight that any proposed implications for large-scale Asian winter monsoon coupling should be interpreted with caution. The corresponding passage now reads:

“This pattern suggests a gradual weakening of winter monsoon winds in the late stage of GI-1, a trend that has also been inferred for the East Asian winter monsoon system based on the magnetic and elemental data from the Huguang Maar[25] (Fig. 3), suggesting a coherent evolution of the entire Asian winter monsoon. However, we note that the use of Huguang Maar sediments as a winter monsoon proxy remains debated[26], and proposed implications for large-scale Asian winter monsoon coupling should be interpreted with caution.”

130 – a solar influence has been demonstrated in other South Asian archives, but why only a multi-centennial influence here? Where are the shorter frequencies?

We thank the reviewer for this point. For this analysis we focused on the solar deVries cycle, which has a periodicity of around 210 years. This cycle has been shown to be present in the ice core ^{10}Be-record and tree-ring ^{14}C-records (Adolphi et al., 2014, 2017), whereas higher frequencies in the ice core ^{10}Be-record are increasingly affected by meteoric influences on ^{10}Be transport and deposition, and hence, cannot be attributed to solar activity changes with the same confidence. Furthermore, as noted by the reviewer, a solar influence has also been demonstrated in other South Asian monsoon records (Wang et al., 2005) but also in ice core climate records (Adolphi et al., 2014). And in both cases, this influence is predominantly seen in the frequency range of the deVries cycle. Hence, we decided to focus on this most robust part of the signal, where our interpretation can be most confident.

163 – it is true that winter precip can influence mean annual d18O (or dD) values, but as these represent small contributions to total annual rainfall, large changes in winter precip are required to make a noticeable dent in the overall isotope ratio. Reference 40 suggests winter rains can account for up to 33% in northern India but many parts of India receive a small percentage in winter.

We largely addressed this point in our previous comment and thank the reviewer for highlighting it again. We argue that isotopically depleted winter precipitation did influence the δD signature in northern India and eventually in our record from northeastern Arabian Sea.

164 – These d13C values seem pretty consistent with C3 plants, and yet they are being used to support the conjecture that C4 plants were present in the Indus River catchment. The shifts are also very small (~1‰). a

We respectfully disagree that our $\delta^{13}C$ values solely reflect C3 vegetation. We highlight that plant-wax alkanes are systematically more depleted due to biosynthesis, leading to lower values than TOC isotope composition (Collister et al., 1994). Commonly used end-members are -22 permille for C4 waxes and -35 permille for C3 waxes. Using endmembers of –35‰ for C3 and –22‰ for C4 plants, the observed $\delta^{13}C$ values (min -26.5 and max -23.5) correspond to roughly 65 to 90% C4 plants, demonstrating that even relatively small isotopic shifts can reflect meaningful changes in C4 abundance.

178 – this seems a bit confusing as monsoon rains should have a lower d18O than non-monsoon winter rains.

We thank the reviewer and note that we have addressed this point above. Here, we specifically refer to long-distance winter precipitation sourced from regions such as the Mediterranean, which can be isotopically more depleted than local summer monsoon rainfall in parts of South Asia (Zhu et al., 2023). During periods of reduced seasonality, such winter precipitation can contribute disproportionately to the integrated δD signal, partially compensating for decreases in summer monsoon rainfall without implying a reversal of the typical monsoon–winter $\delta^{18}O$ relationship.

288 – The chronology is explained using two sentences in the Methods. The first is "The first-order age model for the entire 20.2 m SO130-289KL core is based on 20 accelerator mass spectrometry 14C dates and cross-correlation to radiocarbon dated constrained by 7 14C dates." I've never read a manuscript where no further mention of radiometric dates is made beyond stating they were obtained. Are they robust? Which lab did the work? What materials were dated? Do they fall in stratigraphic order? Where any exlcluded from consideration? If so, why? It's also unclear what is meant by what appears to be the incomplete phrase "and cross-correlation to radiocarbon dated constrained by 7 14C dates". Is there a word missing after dated? How do the 7 14C dates differ from 20 AMS dates?

We realize that it was not clearly explained that the chronology was previously robustly established. We have now corrected this and directly stated that the chronology was previously established in Deplazes et al. (2013).

Reviewer #2 (Remarks to the Author):

This is a promising high-quality paper with impressive methodological advancements and high-quality data.

We thank the reviewer for this positive assessment of our work and for recognizing the methodological advances and data quality presented in this study.

Reviewer #3 (Remarks to the Author):

The manuscript submitted by Obreht et al. for publication in Nature Geoscience presents a unique and exceptionally valuable set of ultra–high-resolution data derived from laminated sediments of the northern Arabian Sea. These records are used to investigate South Asian monsoon dynamics at an unprecedented temporal resolution across the GI-1 interval. The study places particular emphasis on

the South Asian winter monsoon, which has received far less attention than the summer monsoon due to the dominance of the latter in most sedimentary and speleothem archives.

The manuscript is clear and well written. The originality of the data set, together with its innovative use to decipher decadal- to millennial-scale variability in past monsoon systems, strongly supports publication in a journal such as Nature Geoscience. I have only a limited number of relatively minor comments and questions, which should be straightforward for the authors to address.

We are very grateful to Reviewer 3 for their constructive, detailed, and positive evaluation of our work. We sincerely appreciate the time and effort invested in improving our manuscript through the many helpful comments provided.

Line 41 – The statement is not fully correct. Most aeolian dust deposited over the Arabian Sea originates from the Arabian Peninsula and adjacent northwestern regions (Iran–Pakistan) and is transported primarily by northerly to northwesterly winds, often above the summer monsoon layer. These include Shamal-type flows and other dry continental air masses. In contrast, low-level southwest monsoon winds are humid and oceanic in origin and therefore play a secondary, mainly redistributive role rather than acting as the primary dust source.

Thank you for this comment. We have removed the section related to dust transport. Although we fully agree with the reviewer's explanation, we chose not to elaborate further in order to maintain the conciseness of the introduction, as dust transport is not the primary focus of our study.

Line 45 – The authors should already mention here that marine sedimentary records of paleo-monsoon from the Arabian Sea are largely dominated by the South Asian Summer Monsoon (SASM) signal (productivity-related records, upwelling).

Done

Figure 1 – The authors should specify the altitude level at which the back trajectories were computed (e.g., 850 hPa, which is traditionally used for monsoon wind analyses).

We have included this information in the Method section. Analyses were conducted on 850 hPa.

In addition, the rationale for selecting an inland reference point for back-trajectory calculations is unclear. Although backtracking from this location highlights the summer circulation pattern, it is less effective in illustrating the northeast wind regime over the Arabian Sea. A reference point located near the northeastern boundary of the Arabian Sea might have provided a more representative depiction of winter circulation. If the inland position was chosen to characterize moisture transport within the Indus catchment and its relevance to the Bittoo and Timta cave records, this motivation should be explicitly stated in the manuscript. Incidentally, enlarging the map would also allow the authors to indicate the locations of the cave and maar records used in Figure 3.

Our rationale for the selected area for HYSPLIT was to have a better representation of Indus Middle catchment. We selected area around Multan as the HYSPLIT receptor because it better represents the integrated continental air-mass history over the Indus Basin, downstream of the major tributary confluences but upstream of coastal marine influences. Since our objective is to link land-surface hydrology, vegetation, and isotope generation within the basin, the inland location provides a more representative characterization of the terrestrial source region. For comparison, back trajectories computed at the Indus river mouth show a similar pattern (please see Fig. R2 below), showing that the selection of more inland location is not misleading. As a clarification, the northeast wind regime over the Arabian Sea does not appear in these back trajectories because it does not contribute to precipitation. Following reviewer suggestions, we have explained the selection of the location in the figure caption (lines 267-268) and Method chapter (lines 470-472), and also enlarged the map to include other records discussed in the manuscript.

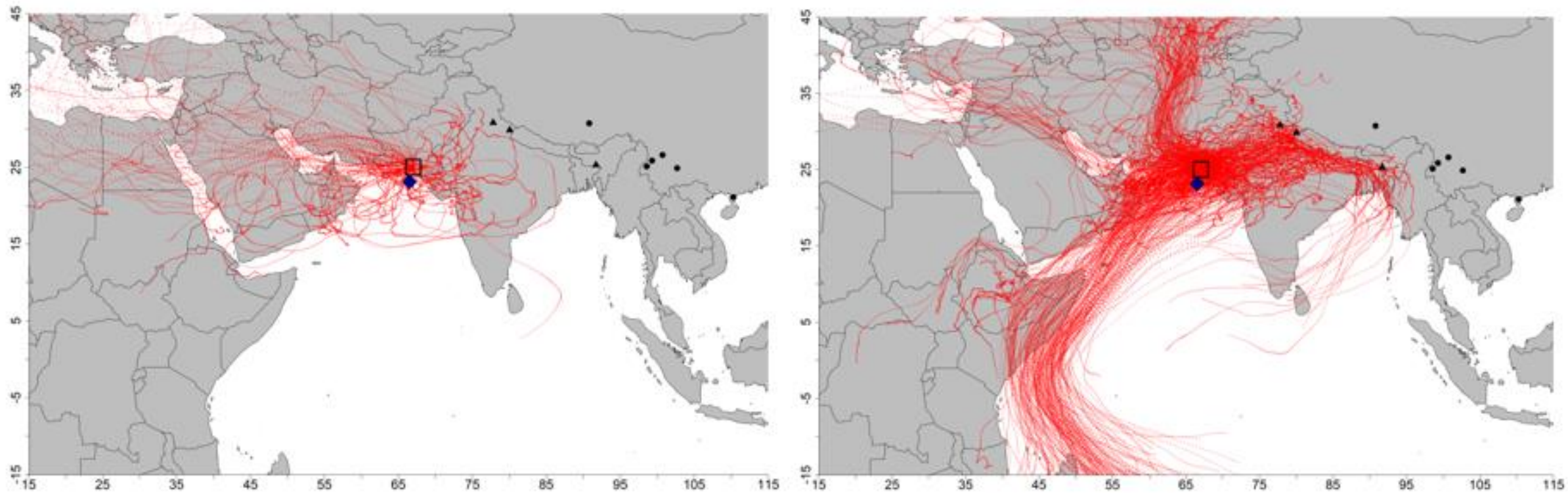

Figure R2. Seasonal back trajectories for the Karatschi region (Indus River delta region; 24-26°N, 66-68°E, trajectories: 25°N, 67°E). Left: Plot of the back trajectories for 240 h during winter. Right: Plot of the back trajectories for 240 h during summer.

We also extended the map to include the caves and maar lakes from Fig. 3 and Extended Data 7. We removed the schematic representation of the ITCZ and monsoon winds from the current image for clarity, since the version with locations had become too cluttered.

Line 54 – The authors correctly describe the studied interval as a natural experiment for investigating the evolution of the South Asian Monsoon (SAM) system during abrupt climate changes. However, they do not sufficiently emphasize here that these rapid climate events occurred within a specific climatic context, namely increasing Northern Hemisphere summer insolation, the onset of the last major deglaciation, and high-amplitude millennial-scale climate oscillations characteristic of glacial conditions. While the limited contextualization at the beginning of the paper does not constitute a strong weakness — given that the authors aim primarily to provide high-resolution insights into monsoon dynamics and not an analogue for modern warming — the broader climatic framework should nevertheless be more clearly articulated from the start.

We explained the background conditions better. Please see lines 51-53.

Line 73 – The authors report using the Uk37 alkenone unsaturation index and the GDGT-based Crenarchaeol–Caldarchaeol Tetraether (CCaT) index as two independent SST proxies. However, they later acknowledge that CCaT is influenced by GDGT cyclization, which responds to oxygen availability and OMZ intensity associated with upwelling in the western Arabian Sea. I suggest that (1) the authors explicitly state from the outset that CCaT is affected by cyclization in this region (with a brief one-sentence explanation of cyclization), and (2) directly reference previous work (e.g., Napier et al.) demonstrating that CCaT at this site is better interpreted as a proxy for oxygenation and, by extension, productivity rather than SST.

Done. Please see lines 74-77.

Accordingly, the raw CCaT index should be better plotted in Figure 2, as is done in Figure 3, instead of presenting CCaT-derived temperatures.

We agree with this suggestion. However, in order to demonstrate that SST estimates are not reliable, we now provide the CCaT-derived SST estimates as an Extended Data 1, together with other relevant temperature records, as alkenone SST and seasonal Greenland ice core temperature data-model reconstruction (see answer below).

Line 105 – Overall, the deglacial CCaT record aligns well with Greenland ice-core $\delta^{18}O$ (Fig. 2), except toward the end of the sequence. Specifically, CCaT declines from the GI-1b/GI-1a transition upward — consistent with the reflectance record and alkenone-based SST— whereas Greenland $\delta^{18}O$ continues to rise before decreasing later during GI-1a. Moreover, the transition to GS-1 corresponds to a sharp decrease in CCaT, with an amplitude comparable to the CCaT increase observed at the HS1–GI-1

transition. In contrast, Greenland $\delta^{18}O$ exhibits a more gradual and lower-amplitude change at the onset of GS-1, unlike the abrupt and large-amplitude shift at the HS1–GI-1 transition. An age-model issue to explain a possible mismatch between the two records seems unlikely, as the SO130-289KL chronology was fine-tuned to Greenland ice-core records. Could the authors provide an explanation for the apparent anti-correlated behavior between CCaT and Greenland $\delta^{18}O$ during early GI-1a, as well as for the discrepancy in signal amplitude?

We thank the reviewer for their insightful comment. We agree that an age-model issue may appear unlikely given that the SO130-289KL chronology was fine-tuned to the Greenland ice-core record using sediment reflectance (L*). However, precisely because the age model is tuned to Greenland $\delta^{18}O$, detailed phase relationships between CCaT and Greenland $\delta^{18}O$, particularly within short intervals such as early GI-1a, cannot be interpreted independently of the tuning procedure. The tuning point within GI-1b (at 3.1 m) was aligned to the $\delta^{18}O$ minimum at 13,211 years b2k. In addition, an abrupt decrease in L* at 2.89 m was tuned to the onset of GS-1 (12,892 years b2k). Importantly, the formal onset of GS-1 in the Greenland record is defined by an abrupt change in deuterium excess, whereas the associated decrease in $\delta^{18}O$ occurs more gradually over approximately 100 years. It is therefore possible that the abrupt L* decrease corresponds not to the very onset of GS-1, but to more negative $\delta^{18}O$ values reached slightly later within GS-1. Small shifts in the placement of these tie-points can influence the apparent phasing during early GI-1a.

While such retuning can reduce the apparent offset, we deliberately refrained from modifying the established chronology because we do not provide independent chronological constraints that would justify a revision. The current age model has been widely used, and further adjustments without additional dating control would reduce comparability with previously published work. Consequently, any subtle anti-phasing or mismatches during early GI-1a should be viewed in light of the tuning approach and the specific selection of tie-points, rather than interpreted as evidence for a mechanistic decoupling.

Regarding the difference in signal amplitude, we acknowledge that some features of the records are not fully consistent. However, we demonstrate that CCaT record shows substantially better agreement with reconstructed data–model–derived summer (JJA) Greenland temperatures than with annual or winter estimates (Buizert et al., 2018) (Extended Data 1). These datasets were not included in the initial manuscript because comparisons between proxy records and proxy–model products can be difficult to compare, nonetheless, we realize that their inclusion improves the clarity of the seasonal interpretation.

Line 119 – Typographical error: “late stage of GS-1” should read “late stage of GI-1”.

Thank you, corrected.

Line 125 – Wavelet spectral analysis indicates a significant in-phase relationship between SST and CCaT on multidecadal timescales during GI-1a and GI-1b. This covariance is also evident from visual inspection and resembles the behavior observed during GI-1d, where decreases (increases) in SST coincide with decreases (increases) in CCaT. Does this imply that SASM and SAWM wind intensities co-varied during these intervals?

This is a valuable observation. The observed proxy covariance would, in principle, support out-of-phase variability of SASM and SAWM wind intensities. However, our interpretation in lines indicated by reviewer is restricted to multidecadal variability. The lack of consistent periodicities does not indicate a commonly known forcing at multidecadal timescales. Thus, we cannot rule out that during this interval, CCaT could have been more strongly affected by thermal forcing on multidecadal timescales, while remaining primarily controlled by oxygenation changes on longer timescales. Such scale-dependent proxy behavior has been reported previously in work from our group on Mediterranean sapropel proxy formation (Obreht et al., 2025). Given this potential complexity in the environmental controls on CCaT, and to avoid overinterpretation, we refrain from making a definitive statement regarding coupled

SASM–SAWM wind variability for this interval (lines 131-136). However, at centennial and longer timescales, the covariance observed during GI-1a–b and GI-d, also evident visually, as noted by the reviewer, we interpret as out-of-phase behavior of SASM and SAWM.

Line 133 – The authors conduct cross-wavelet analyses between Arabian Sea alkenone-based SST, CCaT, and Greenland ice-core 10 Be flux to test whether the 200–250-year periodicity could be linked to solar variability. They report a significant in-phase relationship within this band. Is this zero phase relationship the principal argument for a solar forcing mechanism? Given that the SO130-289KL chronology is tuned to Greenland ice-core records, lead–lag relationships between NGRIP and marine proxies cannot be meaningfully interpreted.

We agree that the exact phase relationship between our proxies and the Greenland ice-core record is strongly influenced by the tuning of the SO130-289KL chronology to the Greenland $\delta^{18}O$ record. Consequently, lead–lag relationships between NGRIP and the alkenone-based SST or CCaT are not meaningful with respect to the $\delta^{18}O$ record used for tuning. However, it is important to emphasize that the ^{10}Be flux record was not used in the tuning procedure. Therefore, the observed in-phase relationship between our marine records and the Greenland ^{10}Be flux within the 200–250-year band is independent of the chronological alignment to $\delta^{18}O$, assuming the overall alignment is correct. In other words, this comparison is only feasible because our record was tuned to the Greenland chronology, but to $\delta^{18}O$ as an independent proxy from ^{10}Be. Without this tuning, age uncertainties would be too large to allow any robust assessment of the phase relationship. In this context, the persistence of a coherent phase relationship throughout the investigated interval provides supportive evidence for a potential link to solar variability. The stability of this phase relationship over time further strengthens the argument that the observed coherence reflects a genuine connection rather than an artifact of tuning.

Line 160 – The authors state that δD values between –155‰ and –136‰ reflect precipitation dynamics within the Indus River catchment, the primary source of leaf-wax n-alkanes. However, Figure 3 shows δD values ranging only from approximately –10‰ to –50‰. This discrepancy should be clarified.

Thank you for indicating this! This is indeed a mistake, as we here referred to non-corrected raw values. This is now corrected.

Moreover, δD values do not unambiguously distinguish wetter interstadial from stadial conditions, as GS-1 values overlap substantially with those from the GI-1 interval.

We deleted: "indicating wetter interstadial than stadial periods but with relatively large fluctuations during the GI-1 (Fig. 3)". We address that reviewer's comment in more details in lines 195–205.

Line 169 – Typographical error: "increase in southeast SASM winds" should read "increase in southwest SASM winds".

Thank you, corrected.

Line 174 – The authors describe a regime shift beginning in GI-1b, characterized by concurrent decreases in $\delta^{13}C$ and δD. However, in my view, the records instead appear to display a broader-scale trend of progressively decreasing $\delta^{13}C$ and δD from HS1 to GS-1, upon which two centennial- to millennial-scale oscillations during GI-1 are superimposed. If confirmed by higher-resolution data, this long-term evolution would suggest a progressive response to gradual forcing mechanisms, such as increasing insolation and/or the ongoing deglaciation. Notably, a trend toward lighter $\delta^{13}C$ values in n-C_{31} alkanes—implying an increased contribution of C_4 plants—would not be straightforwardly compatible with a global increase in insolation-driven seasonality. As pointed out by the authors (ie line 193), an important component of the δD decrease may instead reflect the progressively increasing influence of isotopically depleted Mediterranean-sourced winter moisture during the last deglaciation.

The authors are encouraged to discuss the possibility and underlying mechanisms of a long-term trend in δD and $\delta^{13}C$ over the full duration of the records.

We thank the reviewer for this insightful comment and fully agree with the point raised. We recognize that this aspect was not sufficiently discussed in the previous version of the manuscript, as we also consider it important. We have now incorporated the reviewer's suggestions into the revised discussion, particularly in lines 191–195. In the revised text, we argue that GS-1 was also characterized by increased winter precipitation, implying that the δD signal during this interval was more strongly biased toward winter-derived moisture. This interpretation helps to clarify the long-term trends observed in the isotope records.

Line 197 – I am not convinced by the strategy adopted by the authors. While alkenone-derived temperatures represent robust, well-calibrated SST estimates, their empirical translation into winter wind intensity is far less secure. Furthermore, the authors hypothesize —based on a single published study— that CCaT variability is primarily controlled by GDGT cyclization and therefore does not robustly record temperature, but instead reflects changes in OMZ oxygenation and, by extension, productivity. Even if this interpretation is correct, CCaT would remain a highly indirect and non-calibrated proxy that is only loosely related to summer wind intensity. Consequently, any attempt to quantify the relative strength of SASM versus SAWM by differencing these proxies appears questionable.

We thank the reviewer for this important comment and agree that our previous wording overstated the quantitative implications of the ΔSST_{norm} approach. We have therefore moved this section to the Supplementary Discussion and substantially toned down its interpretation. In the revised version, we explicitly clarify that ΔSST_{norm} is not used as a quantitative measure of SASM versus SAWM strength, but rather as a qualitative and heuristic index of annual monsoon strength. However, given the close correspondence between the normalized SST difference and the isotope-based endmember framework for precipitation seasonality, we retain the comparison as supportive evidence, while clearly limiting its scope to a conceptual and qualitative context.

References

Adolphi, F., Bronk Ramsey, C., Erhardt, T., Edwards, R. L., Cheng, H., Turney, C. S. M., Cooper, A., Svensson, A., Rasmussen, S. O., Fischer, H., & Muscheler, R. (2018). Connecting the Greenland ice-core and U/Th timescales via cosmogenic radionuclides: Testing the synchroneity of Dansgaard–Oeschger events. *Climate of the Past*, *14*(11), 1755–1781. https://doi.org/10.5194/cp-14-1755-2018

Adolphi, F., Muscheler, R., Friedrich, M., Güttler, D., Wacker, L., Talamo, S., & Kromer, B. (2017). Radiocarbon calibration uncertainties during the last deglaciation: Insights from new floating tree-ring chronologies. *Quaternary Science Reviews*, *170*, 98–108. https://doi.org/10.1016/j.quascirev.2017.06.026

Adolphi, F., Muscheler, R., Svensson, A., Aldahan, A., Possnert, G., Beer, J., Sjolte, J., Björck, S., Matthes, K., & Thiéblemont, R. (2014). Persistent link between solar activity and Greenland

climate during the Last Glacial Maximum. *Nature Geoscience*, *7*(9), 662–666. https://doi.org/10.1038/ngeo2225

Alfken, S., Wörmer, L., Lipp, J. S., Wendt, J., Schimmelmann, A., & Hinrichs, K.-U. (2020). Mechanistic Insights Into Molecular Proxies Through Comparison of Subannually Resolved Sedimentary Records With Instrumental Water Column Data in the Santa Barbara Basin, Southern California. *Paleoceanography and Paleoclimatology*, *35*(10), e2020PA004076. https://doi.org/10.1029/2020PA004076

Buizert, C., Keisling, B. A., Box, J. E., He, F., Carlson, A. E., Sinclair, G., & DeConto, R. M. (2018). Greenland-Wide Seasonal Temperatures During the Last Deglaciation. *Geophysical Research Letters*, *45*(4), 1905–1914. https://doi.org/10.1002/2017GL075601

Collister, J. W., Rieley, G., Stern, B., Eglinton, G., & Fry, B. (1994). Compound-specific δ 13C analyses of leaf lipids from plants with differing carbon dioxide metabolisms. *Organic Geochemistry, Compound-Specific Isotope Analysis in Biogeochemistry and Petroleum Research*, *21*(6), 619–627. https://doi.org/10.1016/0146-6380(94)90008-6

Deplazes, G., Lückge, A., Peterson, L. C., Timmermann, A., Hamann, Y., Hughen, K. A., Röhl, U., Laj, C., Cane, M. A., Sigman, D. M., & Haug, G. H. (2013). Links between tropical rainfall and North Atlantic climate during the last glacial period. *Nature Geoscience*, *6*(3), 213–217. https://doi.org/10.1038/ngeo1712

Hughen, K. A., & Heaton, T. J. (2020). Updated Cariaco Basin 14C Calibration Dataset from 0–60 cal kyr BP. *Radiocarbon*, *62*(4), 1001–1043. https://doi.org/10.1017/RDC.2020.53

Hughen, K. A., Overpeck, J. T., Lehman, S. J., Kashgarian, M., Southon, J. R., & Peterson, L. C. (1997). A New 14C Calibration Data Set for the Last Deglaciation Based on Marine Varves. *Radiocarbon*, *40*(1), 483–494. https://doi.org/10.1017/S0033822200018361

Hughen, K., Southon, J., Lehman, S., Bertrand, C., & Turnbull, J. (2006). Marine-derived 14C calibration and activity record for the past 50,000 years updated from the Cariaco Basin. *Quaternary Science Reviews, Critical Quaternary Stratigraphy*, *25*(23), 3216–3227. https://doi.org/10.1016/j.quascirev.2006.03.014

Hunt, K. M. R., Baudouin, J.-P., Turner, A. G., Dimri, A. P., Jeelani, G., Pooja, Chattopadhyay, R., Cannon, F., Arulalan, T., Shekhar, M. S., Sabin, T. P., & Palazzi, E. (2025). Western disturbances and climate variability: A review of recent developments. *Weather and Climate Dynamics*, *6*(1), 43–112. https://doi.org/10.5194/wcd-6-43-2025

Jing, Z., Liu, Z., Zhang, S., Wen, Q., He, C., Bao, Y., & Yu, W. (2025). Precipitation oxygen isotope variability across timescales in East Asia records two sub-processes of summer monsoon system. *Communications Earth & Environment*, *6*(1), 513. https://doi.org/10.1038/s43247-025-02448-1

Müller, P. J., & Fischer, G. (2001). A 4-year sediment trap record of alkenones from the filamentous upwelling region off Cape Blanc, NW Africa and a comparison with distributions in underlying sediments. *Deep Sea Research Part I: Oceanographic Research Papers*, *48*(8), 1877–1903. https://doi.org/10.1016/S0967-0637(00)00109-6

Napier, T. J., Wörmer, L., Wendt, J., Lückge, A., Rohlfs, N., & Hinrichs, K.-U. (2022). Sub-Annual to Interannual Arabian Sea Upwelling, Sea Surface Temperature, and Indian Monsoon Rainfall Reconstructed Using Congruent Micrometer-Scale Climate Proxies. *Paleoceanography and Paleoclimatology*, *37*(3), e2021PA004355. https://doi.org/10.1029/2021PA004355

Obreht, I., Wörmer, L., Varma, D., De Vleeschouwer, D., Schulz, H., Kucera, M., & Hinrichs, K.-U. (2025). Influence of Mediterranean Oceanographic and Environmental Conditions on Molecular Proxy-Based Sea Surface Temperature Reconstructions During the Last Interglacial. *Paleoceanography and Paleoclimatology*, *40*(4), e2024PA004951. https://doi.org/10.1029/2024PA004951

Prahl, F. G., Sparrow, M. A., & Wolfe, G. V. (2003). Physiological impacts on alkenone paleothermometry. *Paleoceanography*, *18*(2). https://doi.org/10.1029/2002PA000803

Rosell-Melé, A., & Prahl, F. G. (2013). Seasonality of UK′37 temperature estimates as inferred from sediment trap data. *Quaternary Science Reviews*, *72*, 128–136. https://doi.org/10.1016/j.quascirev.2013.04.017

Sinnl, G., Adolphi, F., Christl, M., Welten, K. C., Woodruff, T., Caffee, M., Svensson, A., Muscheler, R., & Rasmussen, S. O. (2023). Synchronizing ice-core and U ∕ Th timescales in the Last Glacial Maximum using Hulu Cave ^{14}C and new ^{10}Be measurements from Greenland and Antarctica. *Climate of the Past*, *19*(6), 1153–1175. https://doi.org/10.5194/cp-19-1153-2023

Wang, Y., Cheng, H., Edwards, R. L., He, Y., Kong, X., An, Z., Wu, J., Kelly, M. J., Dykoski, C. A., & Li, X. (2005). The Holocene Asian Monsoon: Links to Solar Changes and North Atlantic Climate. *Science*, *308*(5723), 854–857. https://doi.org/10.1126/science.1106296

Zhu, H., Zhu, L., Luo, L., & Li, J. (2023). Seasonal Variations of Modern Precipitation Stable Isotopes over the North Tibetan Plateau and Their Influencing Factors. *Water*, *16*(1). https://doi.org/10.3390/w16010150

REVIEWER COMMENTS:

Reviewer #1 (Remarks to the Author):

I reviewed an earlier version of this manuscript and had several concerns. The authors have done a fine job of addressing the majority of these and I feel that this is a sufficiently well-constructed and innovative study to merit publication in Nature Geoscience.

We sincerely thank the reviewer for the positive assessment of our revised manuscript and again thank them for their constructive previous comments, which have contributed substantially to the improvement and strengthening of the manuscript.

The only substantive issue remaining is the decision to use end-member rather than mean d13C values for C3 and C4 vegetation (see line 175). This decision seems to have been made to fit the data to the interpretation rather than the other way around and as such, it undermines an otherwise solid paper.

The $\delta^{13}C$ values of plant waxes in our study range between −26.5 and −23.5 ‰ and are thus indicative of a strong contribution of C_4 vegetation. Plant-wax *n*-alkanes are systematically depleted in ^{13}C relative to bulk plant organic matter due to biosynthetic fractionation, resulting in lower $\delta^{13}C$ values (Collister et al., 1994; Chikaraishi et al., 2004). Commonly found end-members for $\delta^{13}C$ values of plant waxes from C3 and C4 vegetation are -35 and -22 ‰VPDB, respectively (e.g. Castañeda et al., 2009; Collins et al., 2011, 2013; Garcin et al., 2014; Schefuß et al., 2003a, 2003b, 2005). A further illustrative example demonstrating this offset in sediments from the Arabian Sea is provided by Feakins et al. (2020), who show persistent depletion of *n*-alkanes relative to bulk organic matter in the same record. Therefore, our $\delta^{13}C$ values of plant waxes of -26.5 to -23.5 ‰VPDB are thus indeed indicating a major C_4 plant contribution. This distinction is now explicitly clarified in the revised manuscript (lines 176–177).

I include a few very minor word-smithing suggestions below.

We appreciate their effort in identifying minor typographical issues and providing helpful suggestions.

38 – please reword “the poleward of equator”

Done

41 – perhaps “drier” since part of the point is that some precip is associated with the SAWM

Done

44 – “increased”?

Done

48 – “sparsity”?

Done

50-57 – “abrupt” is used a lot here. Reword?

Done

67 – no comma after cold

Done

78 – proximal instead of close-by?

Done

116 – trends?

Done

129-136 – “multidecadal” is used a lot here. Reword?

Done

141 – Therefore?

Done

147-with the 10Be?

Done, also corrected in lines 141, 144 and 148.

228 – precipitation, but similar to?
Done

Reviewer #3 (Remarks to the Author):

In my view, the authors have responded satisfactorily to my questions and comments and have improved the quality of the manuscript compared to its initial version. I therefore support the publication of this work in Nature Geoscience.
We greatly appreciate the reviewer’s positive assessment of the revised manuscript and for acknowledging the improvements made in response to their feedback in the previous round of revision; we sincerely thank them for their constructive comments, which have helped to strengthen the quality of the work.

References:

Castañeda, I. S., Mulitza, S., Schefuß, E., Lopes dos Santos, R. A., Sinninghe Damsté, J. S., & Schouten, S. (2009). Wet phases in the Sahara/Sahel region and human migration patterns in North Africa. *Proceedings of the National Academy of Sciences*, *106*(48), 20159–20163. https://doi.org/10.1073/pnas.0905771106

Chikaraishi, Y., Naraoka, H., & Poulson, S. R. (2004). Hydrogen and carbon isotopic fractionations of lipid biosynthesis among terrestrial (C3, C4 and CAM) and aquatic plants. *Phytochemistry*, *65*(10), 1369–1381. https://doi.org/10.1016/j.phytochem.2004.03.036

Collins, J. A., Schefuß, E., Heslop, D., Mulitza, S., Prange, M., Zabel, M., Tjallingii, R., Dokken, T. M., Huang, E., Mackensen, A., Schulz, M., Tian, J., Zarriess, M., & Wefer, G. (2011). Interhemispheric symmetry of the tropical African rainbelt over the past 23,000 years. *Nature Geoscience*, *4*(1), 42–45. https://doi.org/10.1038/ngeo1039

Collins, J. A., Schefuß, E., Mulitza, S., Prange, M., Werner, M., Tharammal, T., Paul, A., & Wefer, G. (2013). Estimating the hydrogen isotopic composition of past precipitation using leaf-waxes

from western Africa. *Quaternary Science Reviews*, *65*, 88–101. https://doi.org/10.1016/j.quascirev.2013.01.007

Collister, J. W., Rieley, G., Stern, B., Eglinton, G., & Fry, B. (1994). Compound-specific δ 13C analyses of leaf lipids from plants with differing carbon dioxide metabolisms. *Organic Geochemistry, Compound-Specific Isotope Analysis in Biogeochemistry and Petroleum Research*, *21*(6), 619–627. https://doi.org/10.1016/0146-6380(94)90008-6

Feakins, S. J., Liddy, H. M., Tauxe, L., Galy, V., Feng, X., Tierney, J. E., Miao, Y., & Warny, S. (2020). Miocene C4 Grassland Expansion as Recorded by the Indus Fan. *Paleoceanography and Paleoclimatology*, *35*(6), e2020PA003856. https://doi.org/10.1029/2020PA003856

Garcin, Y., Schefuß, E., Schwab, V. F., Garreta, V., Gleixner, G., Vincens, A., Todou, G., Séné, O., Onana, J.-M., Achoundong, G., & Sachse, D. (2014). Reconstructing C3 and C4 vegetation cover using *n*-alkane carbon isotope ratios in recent lake sediments from Cameroon, Western Central Africa. *Geochimica et Cosmochimica Acta*, *142*, 482–500. https://doi.org/10.1016/j.gca.2014.07.004

Schefuß, E., Ratmeyer, V., Stuut, J.-B. W., Jansen, J. H. F., & Sinninghe Damsté, J. S. (2003a). Carbon isotope analyses of *n*-alkanes in dust from the lower atmosphere over the central eastern Atlantic. *Geochimica et Cosmochimica Acta*, *67*(10), 1757–1767. https://doi.org/10.1016/S0016-7037(02)01414-X

Schefuß, E., Schouten, S., Jansen, J. H. F., & Sinninghe Damsté, J. S. (2003b). African vegetation controlled by tropical sea surface temperatures in the mid-Pleistocene period. *Nature*, *422*(6930), 418–421. https://doi.org/10.1038/nature01500

Schefuß, E., Schouten, S., & Schneider, R. R. (2005). Climatic controls on central African hydrology during the past 20,000 years. *Nature*, *437*(7061), 1003–1006. https://doi.org/10.1038/nature03945
